# Synthetic Transcription Amplifier System for Orthogonal Control of Gene Expression in *Saccharomyces cerevisiae*

**DOI:** 10.1371/journal.pone.0148320

**Published:** 2016-02-22

**Authors:** Anssi Rantasalo, Elena Czeizler, Riitta Virtanen, Juho Rousu, Harri Lähdesmäki, Merja Penttilä, Jussi Jäntti, Dominik Mojzita

**Affiliations:** 1 VTT Technical Research Centre of Finland, P.O. Box 1000, FI-02044 VTT, Espoo, Finland; 2 Aalto University, Department of Computer Science, P.O. Box 15400, FI-00076 Aalto, Espoo, Finland; 3 Helsinki Institute for Information Technology HIIT, Department of Computer Science, Aalto University, P.O. Box 15400, FI-00076 Aalto, Espoo, Finland; Oxford Brookes University, UNITED KINGDOM

## Abstract

This work describes the development and characterization of a modular synthetic expression system that provides a broad range of adjustable and predictable expression levels in *S*. *cerevisiae*. The system works as a fixed-gain transcription amplifier, where the input signal is transferred via a synthetic transcription factor (sTF) onto a synthetic promoter, containing a defined core promoter, generating a transcription output signal. The system activation is based on the bacterial LexA-DNA-binding domain, a set of modified, modular LexA-binding sites and a selection of transcription activation domains. We show both experimentally and computationally that the tuning of the system is achieved through the selection of three separate modules, each of which enables an adjustable output signal: 1) the transcription-activation domain of the sTF, 2) the binding-site modules in the output promoter, and 3) the core promoter modules which define the transcription initiation site in the output promoter. The system has a novel bidirectional architecture that enables generation of compact, yet versatile expression modules for multiple genes with highly diversified expression levels ranging from negligible to very strong using one synthetic transcription factor. In contrast to most existing modular gene expression regulation systems, the present system is independent from externally added compounds. Furthermore, the established system was minimally affected by the several tested growth conditions. These features suggest that it can be highly useful in large scale biotechnology applications.

## Introduction

Synthetic biology strives to provide means for designing and building genetic systems that are easier and faster to engineer and, at the same time, allow a predictable and precise control of biological systems. In order to prevent unpredictable interference in the functionality of such systems, the concept of orthogonality is of a great importance. An orthogonal system should provide the designed functions with minimal (or in the best case, absent) cross-talk with the host organism, i.e. the functionality of the system should not be influenced by or it should not influence the host organism in other ways than intended [1]. To make the synthetic biology systems designable, it is necessary that they are composed of modular parts. The modularity and orthogonality aspects are essential for our ability to design, apply or transfer the system components for a wide spectrum of applications and host organisms.

In order to achieve maximal yields in biotechnological production, where the target compound is typically synthetized via a heterologous pathway engineered in a production host, the optimal balance in the expression of the individual genes forming the pathway is essential [2]. Furthermore, in our efforts to establish complex genetic control circuits for cell physiology and performance control, there is a clear demand for well-defined modular gene expression regulation elements [3,4]. For the prokaryotic *Escherichia coli* there exist a large number of studies that have extensively mapped the key factors for efficient gene expression and demonstrated development of complex well-controlled genetic circuits [4–8]. Recent studies in eukaryotic gene expression regulation have provided an increasingly wide spectrum of tools for precise expression control. Typically the identified endogenous or engineered promoters [9–11] provide the main regulatory elements for the control. However, also other means of control have been established, such as engineering of the promoter region nucleosome architecture [12] or modification of the mRNA stability via engineering of the terminator [13] or mRNA 3’-UTR sequence [14].

Several studies have recently reported the characterization and engineering of gene expression regulation systems that make use of heterologous, hybrid-transcription factors composed of independent DNA-binding and gene activation domains [15–20]. Most of these systems are regulated by an externally added compound, such as estradiol, testosterone or doxycycline [10,15,17,21]. Although these studies characterize important tools for proof-of-concept studies or analysis of gene functions, the need to use an inducing agent typically represents a potential hindrance for upscaling to an industrial scale due to increased cost.

Here we describe both experimentally and mathematically an orthogonal, modular expression system that is independent from externally added compound(s) and enables tight control over a wide range of expression levels for multiple genes in *S*. *cerevisiae*.

## Materials and Methods

### Strains and media

*Saccharomyces cerevisiae* CEN.PK113-11C (H3896; MATα, *ura* 3–52 *his*3Δ1 MAL2-8^c^ SUC2) was used as the parental strain. The expression cassettes (Table A in S1 File) were introduced to cells through transformation of the linearized integrative plasmids. In case of the pHIS3i-plasmids the linearization was done by *Nsi*I digestion and in case of the pBID-plasmids by *Eco*RV digestion. Transformations were performed using the standard Li-Acetate protocol. For the experiments presented in section “Core promoter module analysis and engineering of an orthogonal system”, the expression cassette for the sTF42 controlled by the *MET17* promoter was integrated into the *his*3*Δ1* locus in three copies and the pBID1 reporter cassettes were integrated into the *ura3-52* locus in two copies. In the experiments presented in sections “The development of an external-signal-independent system” and “Regulated sTF42 increases dynamic range of the output signal”, the expression cassettes for the sTFs with either a weak constitutive *TDH3* core promoter or an inducible *MET17* promoter were integrated into the *his*3*Δ1* locus in single copy and the pBID2 reporter expression cassettes were integrated in the *ura3-52* locus in two copies.

For cultivations, 6.7 g/L of yeast nitrogen base (YNB, Becton, Dickinson and Company), synthetic complete amino acid mixture lacking uracil and histidine (SC-HU) supplemented with 20 g/L D-glucose (SCD-HU) was used. Media modifications, such as modified methionine concentration or replacement of D-glucose with D-galactose, are specified in the Results. For the analysis of the functional stability of the expression system, the cultivations were done in either SC medium or YP medium (10 g/L yeast extract; 2 g/L Bacto peptone) supplemented with either 20 g/L D-glucose (SCD or YPD) or 0.5% ethanol (SC-EtOH or YP-EtOH).

### Construction of DNA parts

The detailed description of the construction is provided in the Supplementary methods (in S1 File) and the plasmids used in this study are listed in the (Table A in S1 File). All the constructs generated in this study are available upon request.

### Isolation of sTF16 and electrophoretic mobility shift assay (EMSA)

Open reading frame (ORF) of the sTF16 gene was amplified from the plasmid pHIS3i-(*MET17*p)-sTF16 with primers 1017 and 1018 (Table B in S1 File), introducing *Nde*I and *Sal*I before ATG and stop codons, respectively. The *Nde*I–*Sal*I-digested PCR product was ligated into the *Nde*I–*Sal*I fragment (5251bp) of the bacterial expression plasmid pET26b(+) (Novagen). The sequenced product, pET26b-sTF16-6×HIS, was transformed into the protease deficient *E*. *coli* strain BL21(DE3), allowing IPTG-inducible expression of genes under T7*lac*-promoter. The sTF16-6×HIS protein was purified according to QIAexpressionist manual protocols 9 and 12 (Qiagen). Briefly, cells were grown in 500 ml of 2×YT medium to OD_600_~0.6 at 30°C, IPTG was added to a final concentration of 1 mM, and the cultivation was continued for 3 hours followed by centrifugation, washing and resuspension in 30 ml of lysis buffer with protease inhibitors (Complete, Roche). Lysozyme was then added to 1 mg/ml, the cell suspension was incubated on ice for 30 min, sonicated in four 1-minute sessions with 2 min breaks on ice, and finally centrifuged at 20000 rpm for 20 min. A cleared lysate was incubated with 1 ml of Ni-NTA agarose (Qiagen) for 20 min at 4°C (gentle agitation) prior to loading on a column. The Ni-NTA agarose was washed with the washing buffer and the protein was eluted in 4 × 0.5 ml of the elution buffer. The elution buffer was exchanged on the PD-10 column (BioRad) for the dilution buffer (10 mM Tris; 150 mM KCl; pH = 8.0) and the protein was stored at -80°C in 40% glycerol solution.

The B1, B2, B3, and B4 versions of the LexA-binding site-containing DNA fragments were individually labelled with a red fluorescent dye, Cy-5; to serve as labelled DNA probes in EMSA. Each probe was synthetized from two Cy-5-labelled primers, which were self-annealed and the single-stranded-DNA ends were filled by the DNA polymerase (Phusion, Life Technologies). The B1 version was assembled with primers 1043 and 1044 (Table B in S1 File); the B2 version using primers 1045 and 1046; the B3 version with primers 1047 and 1048; the B4 version using primers 1049 and 1050. The unlabelled competitor DNA probe, containing 4×BS (B1-B2-B3-B4), was obtained by PCR from the pBID2-EP-4 plasmid with primers 1051 and 1052.

The EMSA was performed according to a general protocol [22]. The reactions (15 μl) were assembled on ice, each binding reaction containing: 5 μl of 10× diluted sTF (0.6 mg/ml), 2 μl of unlabelled competitor (4×BS; original concentration 150 ng/μl, 10× diluted, or water in case of controls), 100 ng/μl poly-dIdC (Sigma), 10 mM Tris (pH = 8.0), 150 mM KCl, 0.5 mM EDTA, 0.1% Triton X-100, 12.5% glycerol, 0.2 mM DTT, 0.1 mg/ml BSA, and 0.15 μl of the labelled DNA probe (75 ng/μl each). The binding reactions were incubated at 6°C for 30 min. The gel (18 well, precast, 4–20%, BioRad) was pre-run in 0.5×TBE buffer at 6°C for one hour, samples were loaded and the electrophoresis performed at 190 V for 1.5 hour. After electrophoresis, the gel was transferred into a container with distilled water, briefly washed, enclosed in transparent plastic film, and scanned by the Typhoon Trio Imager (GE Healthcare).

### Cultivations and fluorescence analysis

Pre-cultures were grown for 24–48 hours on the SCD-HU agar plates prior to inoculation of 25 ml of SCD-HU in 100 ml Erlenmeyer flasks to OD_600_ = 0.2. The cultures were grown for 16 hours at 250 rpm and 30°C, centrifuged, washed, and resuspended in 0.5 ml of sterile water. Two hundred μl of the cell suspension was analysed in black 96-well microtiter plate (NUNC) using the Varioskan (Thermo Electron Corporation) fluorimeter. The settings for GFP were 470 nm (excitation) and 535 nm (emission), and for mCherry 587 nm (excitation) and 610 nm (emission), respectively. For normalization of the fluorescence results, the analysed cell-suspensions were diluted 100× and OD_600_ was measured in transparent 96-well microtiter plates (NUNC) using Varioskan (Thermo Electron Corporation).

### Strain and transcription analysis by RT-PCR

The copy number of the expression-cassette genomic insertions in the yeast strains were tested by RT-PCR. Several single colonies were re-streaked from the transformation plate on a new selection agar plate, and incubated at 30°C for 24 hours. Cell biomass of the size of a match head was resuspended in 0.6 ml of TE buffer and combined with 0.6 ml of acid-washed glass beads (Sigma) and 0.6 ml of PCI (50% phenol, 48% chloroform, 2% isoamyl-alcohol) in a 2 ml tube with a screw cap. The cells were disrupted in 2 sessions of vigorous shaking in the Precellys24 homogenizer (Bertin Technologies). The samples were centrifuged at 13000 rpm for 8 min, the resulting supernatant diluted 100× in water, and used as a template for RT-PCR.

To quantify the transcription of the reporter genes, total RNA was isolated with RNeasy Plant Mini Kit (Protocol 1c –mechanical disruption of cells with DNAse on-column treatment; Qiagen) and subsequently used for cDNA synthesis (Transcriptor First Strand cDNA synthesis kit; Roche). The cDNA was diluted 20× in water and used as a template for RT-PCR.

The genomic DNA as well as the cDNA was analysed by RT-PCR in a LightCycler 480 Instrument II (Roche) and the analysis was performed with the accompanying software (Advance Relative Quantification tool). The primers used for the analysis of GFP were 1007 and 1008; for mCherry– 890 and 891; sTF42–806 and 807; and sTF16–971 and 972. In each case, the signal was normalised to that of copy-number/transcription of the *IPP1* gene (primers 484 and 485).

### Mathematical modelling

We developed mechanistic dynamic models in terms of ordinary differential equations for the expression systems using either the methionine induced sTF or the constitutive sTF starting from the biochemical reaction networks described in the Tables C and D (in S1 File), respectively. Although the experimental constructions were bi-directional, leading to the expression of both GFP and mCherry, we chose to include in our model only the part corresponding to mCherry. This reduction does not decrease the predictive power of the model since the reactions leading to the expression of GFP and mCherry were not competitive, sharing only the initiation step, i.e., the binding of the sTF to the sTF-specific binding sites. Both mathematical models were derived by assuming mass-action kinetics for each reaction [23,24]. The main components included in the two models are: polymerase; methionine induced transcription factor (MetTF), which regulates the expression of the synthetic TF (sTF); methionine (met), which sequestrates MetTF and thus hinders the transcription of sTF; the fluorescent protein mCherry; the associated mRNAs (MTFc, MmCherry_c); and genetic elements such as promoter sites (CP for mCherry and DTF for the sTF) and binding boxes for the transcription factors (BTF—the binding site for MetTF, and B—the binding site for sTF). Each reactant is assigned to one of the two compartments explicitly included in the models: the cytoplasm or the nucleus as detailed in the Table E (in S1 File). The reactions included in the two models illustrate different binding/unbinding processes, the translocation of the sTF from cytoplasm to the nucleus, the transcription and translation of sTF and mCherry as well as the degradation of proteins (TFc, TFn and mCherryc) and mRNAs (MTFc and MmCherryc) (see Tables C and D in S1 File). To map the model quantities to the experimental measurements we also considered a scaling factor for mCherry fluorescence levels.

The only difference between the models corresponding to the methionine induced sTF and constitutive sTF systems stands in the sTF transcription, i.e. reactions 2–4 from the Table C (in S1 File) and reaction 2 from Table D (in S1 File), respectively. The kinetic parameters for all the other corresponding reactions were set to be identical in the two models, as detailed in the Table F (in S1 File). The only difference between the models corresponding to the systems using the sTF16 or sTF42 constructs stands in the kinetic rates associated to 2 reactions: i) the association of the polymerase and the sTFs bound to their specific DNA sites, and ii) the degradation rate of the sTF proteins corresponding to the two transcription factors sTF16 or sTF42. The SBML source code of these 4 models was deposited in the BioModels database [25] and assigned the identifiers MODEL1510230001, MODEL1510230002, MODEL1510230004, and MODEL1510230005.

We partitioned the experimental data into a training set and a validation set. When setting up the values for the methionine-induced sTF model, the training set consisted of (i) the sTF16 constructs with 0, 1, 2 and 6 sTF-binding sites from 100 and 200 μM methionine levels, (ii) the sTF16 constructs with 2 and 6 sTF-binding sites from 500 and 1000 μM methionine levels, and (iii) the sTF42 constructs with 1 and 6 sTF-binding sites from 0, 100, 200, 500 and 1000 μM methionine levels. This training data was used to estimate 30 model parameters, see Table F (in S1 File). All the other 41 experimental data sets formed the validation set. For parameter estimation we used simulated annealing [26] as the global optimization function to minimize the sum of squares objective function

$$f(X,\theta )=\sum_{n}{\left(Y_{n}-X_{n}(\theta )\right)}^{2},$$

where *Y*_*n*_ and *X*_*n*_(*θ*) stand for the n-th experimental measurement and the model prediction for the n-th data point, respectively, and *θ* denotes the model parameters. In order to assess the goodness-of-fit between the model predictions and the data we computed the *R*^2^ score, also known as the coefficient of determination. More information about this score is included in the S1 File and the *R*^2^ values obtained for all the considered data sets are reported in the Table G (in S1 File). All model analysis and simulations were done by using the software COPASI [27].

The set of parameter values identified for the methionine-induced system was then introduced in the model corresponding to the constitutive sTF system. Since sTF transcription is the only process that is different in this model when compared with the methionine-induced system, we only needed to estimate the corresponding kinetic parameter. For this we partitioned the experimental data for the constitutive systems into a training set consisting of the constructs with 1, 2 and 6 sTF-binding sites for both sTF16 and sTF42 systems and a validation set consisting of all the other 8 data sets. The obtained set of parameter values was then introduced in the model associated to the system using the sTF16 construct with the pBID2-ED (*DAN1*cp–mCherry), instead of previously used pBID2-EP (*PGK1*cp–mCherry). In this new model, we only needed to re-estimate the kinetic parameters related to the recruitment of polymerase associated with sTF to the core promoter (reaction 7 in Table D in S1 File), while all the other reactions had the same kinetics as in the previous model. For this parameter estimation, we divided the set of associated experimental data (for the system using the strong constitutive sTF16 with pBID2-ED) into a training set and a validation set. The training set consisted of the sTF16 constructs with 1, 2 and 6 sTF-binding sites and the validation set consisted of all the other 4 data sets corresponding to the systems with 0, 3, 4 and 8 sTF-binding sites.

## Results

### The transcription amplifier concept

**Fig 1:**
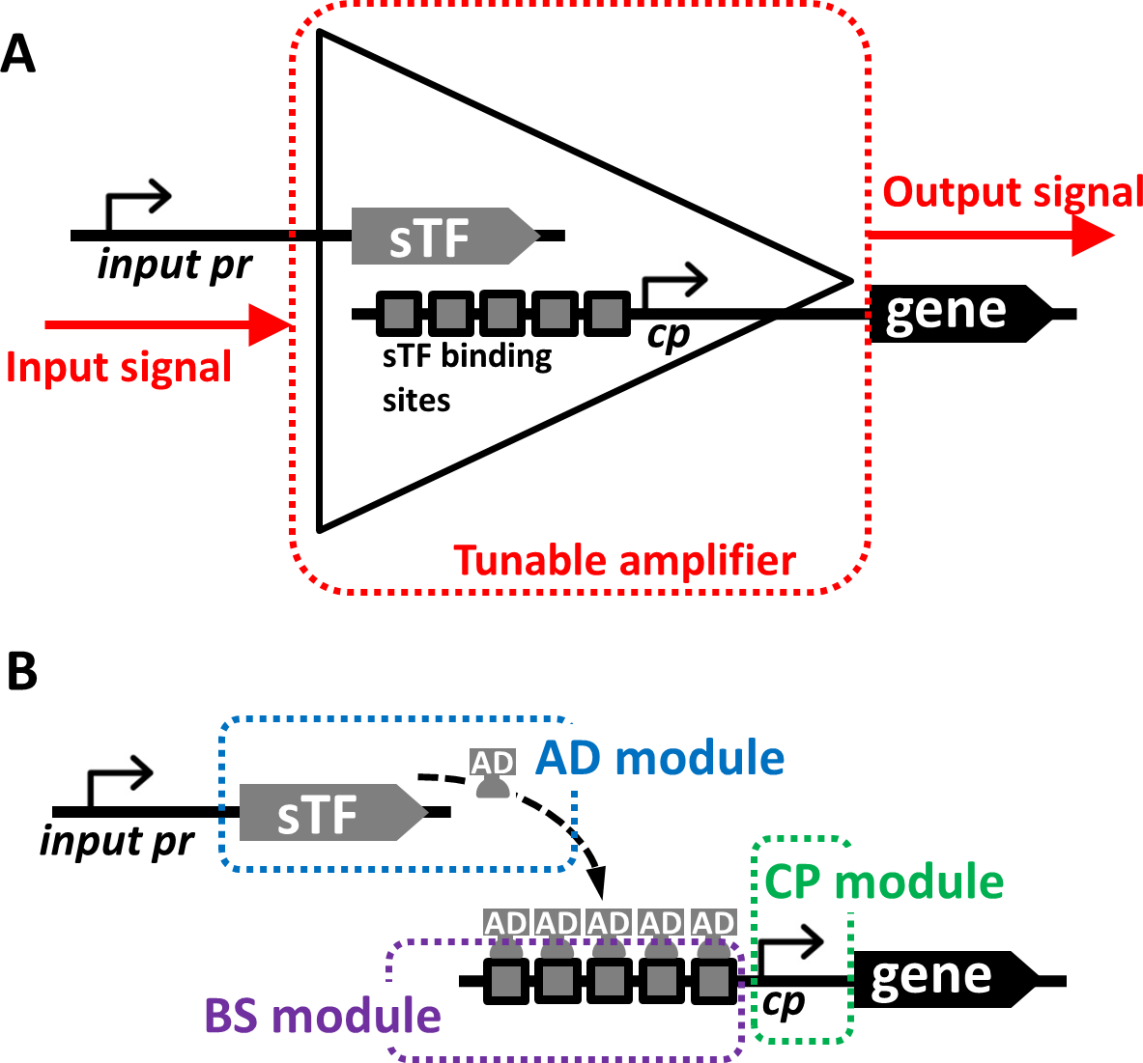
The expression amplifier concept. **A)** The input signal is conveyed to the system in form of an activity of the promoter controlling sTF expression (input pr). This could be a constitutive signal or a signal generated in response to physiological changes in cells, such as metabolic or stress responses, or it can be a signal provided by an engineered synthetic control circuit. The input signal is transformed in a cascade of events including production of the sTF, binding the sTF to its specific sites in the output promoter, assembly of the transcription machinery on the core part of the output promoter (cp), and finally initiation of transcription of target gene. The expression of the target gene is the output signal of the system. **B)** The components, or modules, of the system significantly contribute to the efficiency of the input signal transformation. The amplification of the input signal is achieved by selecting the modules with specific activities providing predictable output signal which is not influenced by the context of the host genetic regulations (the orthogonal system).

We have previously developed an expression system which utilizes a stable genome-integrated expression cassette containing a synthetic transcription factor (sTF) and a synthetic sTF-dependent promoter controlling the transcription [28]. The sTF was a fusion protein composed of the LexA-DNA binding domain, SV40 nuclear localization signal and the B42 activation domain [29], and its expression was under the inducible *MET17* promoter. The sTF-dependent promoter was a modified *GAL1* promoter containing 8 copies of a LexA operator. The system functions as a transcription amplifier (Fig 1A) where the input signal, the availability of methionine, is conveyed to an output signal driving the expression of the target gene. A transcription amplifier can be defined as a genetic system where a weak transcription level (input signal) is amplified in a cascade of steps into a strong transcription output. We identified three principal modules that are essential for the amplification function (Fig 1B) and could be modified in order to modulate signal transduction strength and consequently result in a wide spectrum of expression levels. First, the binding site (BS) module, which is the sTF-specific BS in the upstream activating region of the output promoter. The number of the BS could influence the transcription activity similarly to the known native transcription factor binding sites in the native promoters such as *GAL1*p [30,31] or similarly to previously designed orthogonal systems [10,15,17,21]. Second, the activation domain (AD) module, which is an effector part of the sTF; i.e. the transcription activation domain which confers specific strength. And third, the core promoter (CP) module, which is the core promoter necessary for assembly of the general transcription machinery and for initiation of transcription.

### The BS module–diversification and characterization of the LexA-binding motifs

The previously characterized LexA binding motif has a consensus sequence of a 16 bp palindrome, CTGTATATATATACAG [32]. *E*. *coli* contains a number of genes regulated by the LexA repressor. These genes, as well as the previously used modified *GAL1* promoter with 8 LexA BS, contain a spectrum of sequence modifications deviating from the consensus LexA binding site [32,33]. Based on the native LexA binding sites present in the regulatory region of the *lexA* gene [32] and in the *ColEI* operator [33], we designed versions of the LexA BS with minor sequence modifications (B1-B4; Figure A in S1 File) and tested their binding affinity to purified sTF16 in the electrophoretic mobility shift assay (EMSA) (Figure C in S1 File). The sTF16 used was composed of a LexA DNA binding domain, the Herpes simplex virus transactivation domain (VP16) and a 6×His-tag. The results indicate that all four versions of the BSs bind similarly to the LexA-DNA-binding domain *in vitro* and therefore can also be expected to perform similarly within a synthetic output promoter *in vivo*.

### Core promoter module analysis and engineering of an orthogonal system

**Fig 2:**
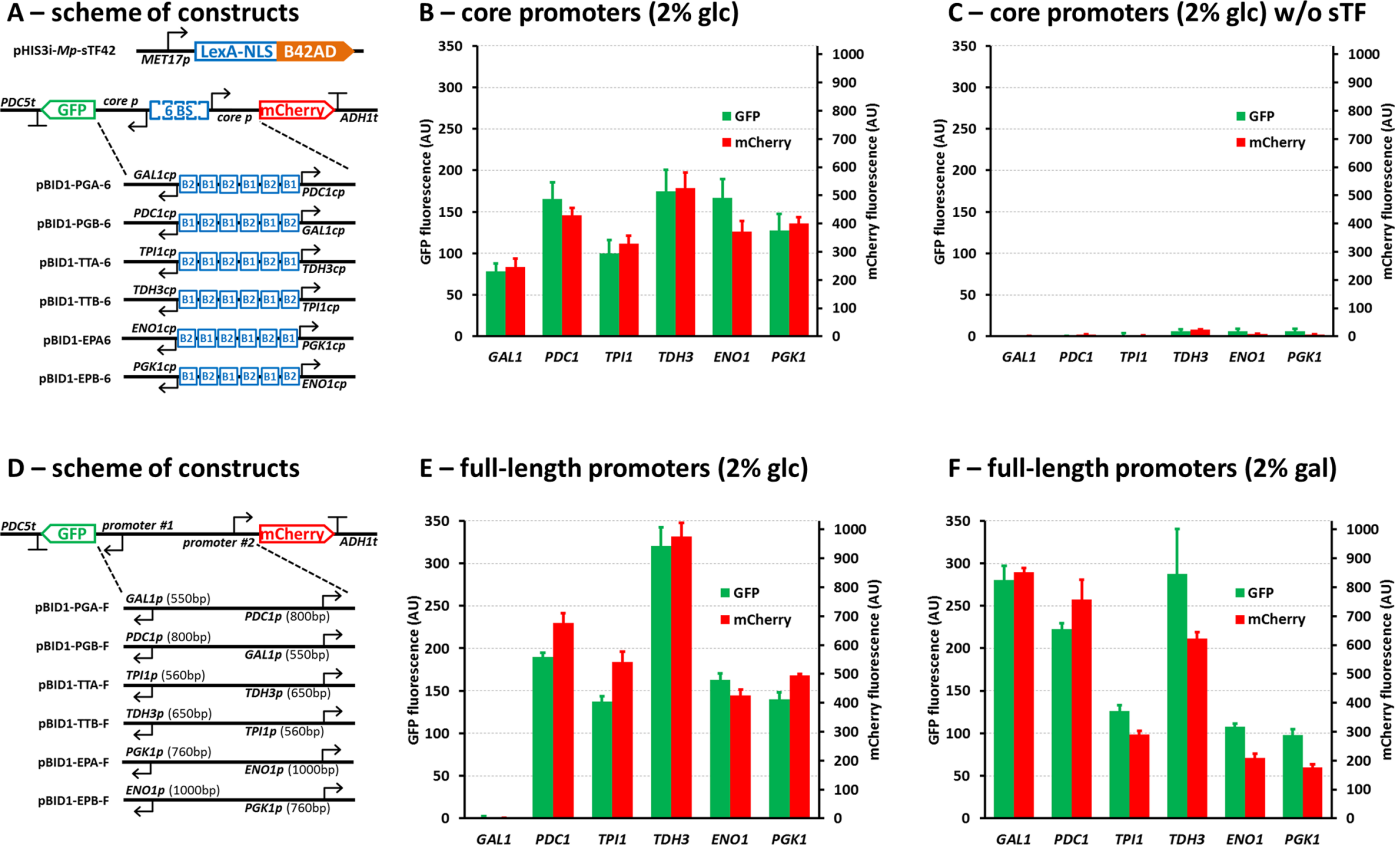
Characterization of the core promoters. **A)** Schemes of the DNA constructs used in B and C. The bidirectional promoters are composed of the outward oriented core promoters of the indicated genes (for the sequence details see Figure B in S1 File) and six LexA-binding sites inserted between the core promoters. **B)** The normalised fluorescence signal of the strains grown in presence of 2% D-glucose and in absence of methionine. The fluorescence signal is shown for each core promoter individually. **C)** Assessment of the background activities of core promoters. The control strains with the same set of the genome-integrated pBID1 reporter expression cassettes as in B, but without the sTF expression cassette. **D)** Schemes of the constructs used in E and F. The reporter cassettes with full-length promoters assembled in bidirectional orientation. **E)** The normalised fluorescence signal of the strains grown in presence of 2% D-glucose and in absence of methionine. **F)** The normalised fluorescence of the strains grown in presence of 2% D-galactose and in absence of methionine. The values represent the averages and the error bars the standard deviations from at least 3 independent experiments.

In order to establish DNA modules with predictable properties for the assembly of the general transcription initiation complex in *S*. *cerevisiae*, we selected six *S*. *cerevisiae* promoters which are commonly used for heterologous gene expression in this host: *GAL1p*, *PDC1p*, *TPI1p*, *TDH3p*, *ENO1p*, and *PGK1p*. With the exception of the *GAL1*p, which is repressed by D-glucose and induced by D-galactose, these promoters are considered strong and constitutive. For each promoter the selected region was about 200 bp upstream of the translation initiation codon ATG (the exact sequence information is shown in Figure B in S1 File). This region should include all the general transcription regulatory motifs, such as TATA-box, and exclude all the specific regulatory motifs, such as UAS sequences (binding sites for native activators and repressors). This part of the promoter DNA is generally referred to as “the core promoter”. We then made use of the modified LexA DNA-binding sites and the core promoters to construct three pairs of synthetic bidirectional promoters containing 6 BSs flanked with two outwards oriented core promoters defining the transcriptional direction. Each bidirectional promoter was then employed to drive expression of GFP or mCherry reporter genes (Fig 2A). This design was based on the assumption that the sTF bound to the promoter does not confer preference in terms of the activation to any specific direction and the directional aspect is defined by the assembly of the general transcription machinery on the core promoter part. The sTF used for transcription activation of these promoters was identical to the previously published version [28], the *MET17p*-controlled LexA-NLS-B42AD (sTF42). However, unlike in the previously published system [28], the sTF is expressed from a separate expression cassette (the sTF and the output (reporter) cassette were integrated in two different genomic loci (*HIS3* and *URA3*, respectively)).

When the system was tested in the medium lacking methionine (to maximize the expression of the sTF), significant expression of both reporters was observed (Fig 2B). The choice of the core promoter had a clear effect on the expression levels of the fluorescent proteins so that, the *GAL1*cp enabled the weakest and the *TDH3cp* the strongest expression. The level of fluorescence varied around 2-fold between the *GAL1cp*- and the *TDH3cp*-containing constructs. To demonstrate the requirement of the sTF for the expression and to assess the level of background activity of the core promoters, we tested strains lacking the sTF-expression cassette for reporter gene activity. Negligible production of the reporters was observed from the core promoters when no sTF was present (Fig 2C).

To compare the activity of the synthetic promoter system to the full-length versions of the selected promoters, analogous pairs of the reporter cassettes were constructed (Fig 2D), introduced to *S*. *cerevisiae*, and tested under the same conditions as the synthetic constructs. In cells grown in D-glucose, the measured GFP and mCherry fluorescence was in the same range with the synthetic constructs, with a notable difference in the case of the *TDH3* and the *GAL1* promoters, where significantly stronger and no expression was observed, respectively (Fig 2E). As expected, when the same strains were grown in the presence of D-galactose, the expression levels for the reporter genes controlled by the *GAL1* promoter showed a dramatic up-regulation reaching similar levels as observed in case of the full-length *TDH3* promoter (Fig 2F).

### The development of an external-signal-independent system

Our goal was to establish a system that would not be dependent on the presence of an external inducer. When a sTF with stronger transcription activation domain, VP16 (sTF16) was expressed under *MET17* promoter in medium lacking methionine (induced condition), a very low growth rate was observed for the yeast strain and the level of fluorescence was not reproducible (data not shown). This indicates that overexpression of VP16 domain is not well tolerated by the strain used. To generate a system where the expression level of a strong sTF would remain at a well-tolerated level under different growth conditions, we designed a new expression cassette for the sTFs. In designing the system we utilized the knowledge from the core promoter tests (Fig 2C), where the *TDH3* core promoter showed low, but consistent expression output above the background. Furthermore, in order to be able to better predict the behaviour of the system, a mathematical model was generated.

**Fig 3:**
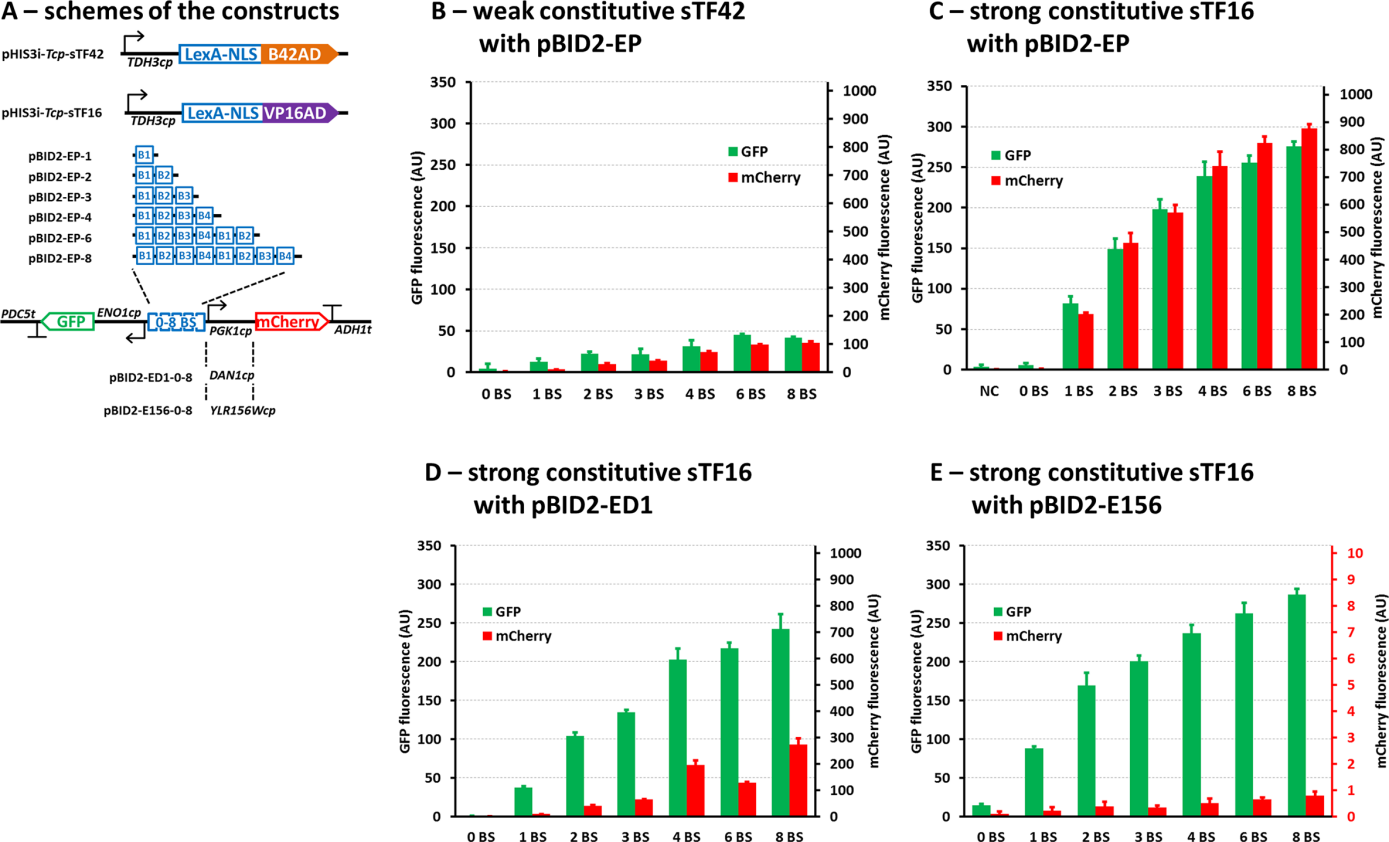
Development of the constitutive system. **A)** Schemes of the DNA constructs used in B–E. The bidirectional promoters are composed of the outwards oriented core promoters of the genes indicated (for the sequence details see Figure B in S1 File) and the varying number of the LexA-binding sites. **B)** The normalised fluorescence of the strains with the weak sTF42 and *ENO1*cp-GFP / *PGK1*cp-mCherry reporter constructs. **C)** The normalised fluorescence of the strains with the strong sTF16 and *ENO1*cp-GFP / *PGK1*cp-mCherry reporter constructs. **D)** The normalised fluorescence of the strains with the sTF16 and *ENO1*cp-GFP / *DAN1*cp-mCherry reporter constructs. **E)** The normalised fluorescence of the strains with the sTF16 and *ENO1*cp-GFP / *YLR156W*cp-mCherry reporter constructs. All the graphs are showing the same range of values on axes depicting the GFP and mCherry fluorescence as in Figs 2 and 6, with exception of the mCherry fluorescence in E, where the range of the axis (in red) was adjusted to visualize negligible fluoresce in these strains. The values represent the averages and the error bars the standard deviations from at least 3 independent experiments.

In order to diversify the output signal strength, a set of bidirectional promoters were constructed where the LexA-binding sites (BS) varied from zero to eight copies. Single integrations of either weak sTF42 or strong sTF16 under the control of the *TDH3*cp, in combination with the genome integrated reporter expression cassettes containing 0 to 8×BS resulted in a wide spectrum of expression levels (Fig 3A–3C). The system controlled by the constitutively expressed weak sTF42 provided a range of low expression levels, where the levels of GFP or mCherry fluorescence correlated with the number of the BSs, ranging from just-above-background values (1×BS; pBID2-EP-1) to approximately 10% of the *TDH3*-full-length promoter output (Fig 3B). On the other hand, the system controlled by the strong sTF16 showed a range of high expression levels from about 20% (1×BS; pBID2-EP-1) to about 93% (8×BS; pBID2-EP-8) of the *TDH3*-full-length promoter output (Fig 3C). Both of these dynamics were very well captured by the mathematical model (Fig 6A and 6B). These results demonstrate that the synthetic expression system enables expression of target genes over a wide dynamic range.

In order to assess the amplification gain of the developed system, the strength of the input signal and the output signal were compared (Figure D in S1 File). This was done by dividing the fluorescence signals obtained in the *TDH3*cp driven expression of either GFP or mCherry (Fig 2C), which should correspond to the expression of the sTFs in the constitutive system, and the fluorescence or either GFP or mCherry obtained in the constitutive system (Fig 3B and 3C). The highest amplification gain was detected in the expression system composed of the sTF16 and the pBID-EP-8, and it was approximately 42× for GFP expression and 38× for mCherry expression (Figure D in S1 File). We also addressed stability of the expression system in diverse growth condition. For this, the transcription level was analysed for the sTF16 and mCherry in defined (SC) or rich (YP) media in presence of either glucose or ethanol during 24 hours of cultivation (Figure E in S1 File). The results indicated that the expression of both genes remained stable in all tested conditions with exception of a minor decline during the exponential growth phase (especially in YPD). Importantly, the ratio of the sTF and the mCherry transcript remained similar in all conditions, and the amplification gain was 73× ± 17 for the conditions corresponding to the fluorescent measurements and 88× ± 31 considering all measured instances.

**Fig 4:**
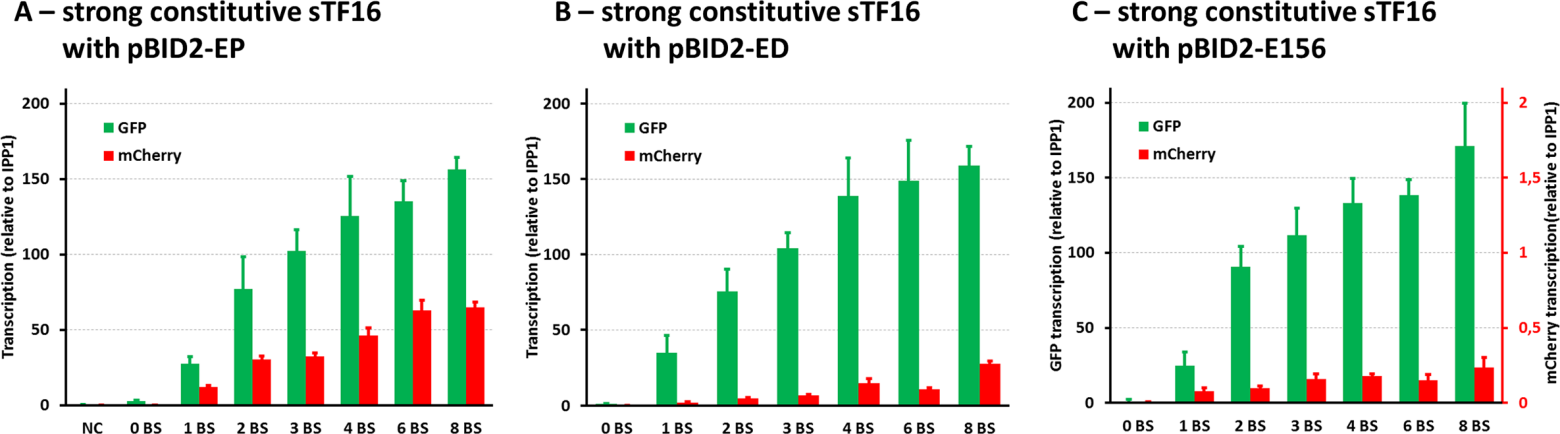
Transcription analysis of different synthetic promoters. **A)** The levels of GFP and mCherry mRNAs corresponding to the strains shown in Fig 4C; the sTF16 and *ENO1*cp-GFP / *PGK1*cp-mCherry **B)** The levels of GFP and mCherry mRNAs corresponding to the strains shown in the Fig 4D; the sTF16 and *ENO1*cp-GFP / *DAN1*cp-mCherry. **C)** The levels of GFP and mCherry mRNAs corresponding to the strains shown in the Fig 4E; the sTF16 and *ENO1*cp-GFP / *YLR156W*cp-mCherry. The values represent fold-difference relative to the *IPP1* mRNA as determined by RT-PCR. All the graphs are adjusted to show the same range of values on x-axes depicting the level of transcription, with the exception of mCherry RNA levels in C, where the range of the axis (in red) was adjusted to visualize negligible mCherry expression in these strains. The values represent the averages and the error bars the standard deviations from at least 4 experimental replicates.

The wide range of expression described above was achieved through the use of two different sTFs. In order to expand the expression range of the system controlled by a single version of sTF, we diversified the CP module of the pBID2-EP cassettes, by introducing weaker core promoters instead of the *PGK1*cp for expression of the mCherry reporter gene. At the same time the expression of GFP was retained under control of the *ENO1*cp (Fig 3A). Two weak promoters were tested, the core promoters of the *DAN1* gene, previously shown to be a weakly expressed gene [17], and the core promoter of the *YLR156W* gene, previously shown to confer negligible expression [34]. The *DAN1* and *YLR156W* core promoters had a significant influence on the expression output, the *DAN1*cp providing moderate expression activation and the *YLR156W*cp providing negligible expression (Fig 3D and 3E). The established gene expression system demonstrated expression output covering virtually whole spectrum of expression levels that are observed for native *S*. *cerevisiae* promoters, ranging from 0 to 93% of the *TDH3* promoter activity (Figs 2B and 4). The additional modulation of the fluorescence levels due to the change of the core promoter from *PGK1*cp to *DAN1cp* is also illustrated by the mathematical model (Fig 6C). However, an unexpected behaviour of the system was observed for pBID-ED-4 and pBID-ED-8, where the mCherry fluorescence was significantly higher compared to the rest of the tested systems with the *DAN1*cp. The model under-estimation indicates that there is an additional effect, presumably by the close proximity of the B4 version of the LexA BS (present on the 3’-end of the BS module in pBID-ED-4 and pBID-ED-8) with the *DAN1*cp, that results in higher than expected expression of mCherry. This hypothesis was tested *in silico* by allowing the models corresponding to these 2 constructs to have different kinetic parameters for the association of polymerase bound to sTF with the core promoter than the other 6 models corresponding to the constructs with 0, 1, 2, 3 and 6 binding sites (Figure G in S1 File). It was observed that including such an assumption in the model significantly improved the fit with the experimental data. To validate that the results obtained by the fluorescent measurements are a true representation of the functionality of the promoter modules, we also assayed transcriptional levels of GFP and mCherry mRNA. The levels of the mRNAs relative to the *IPP1* control mRNA show a good correlation with the amount of GFP and mCherry fluorescence (Fig 4). Taken together, these results verify predictable and reliable operation of the established gene expression system.

### Regulated sTF42 increases dynamic range of the output signal

**Fig 5:**
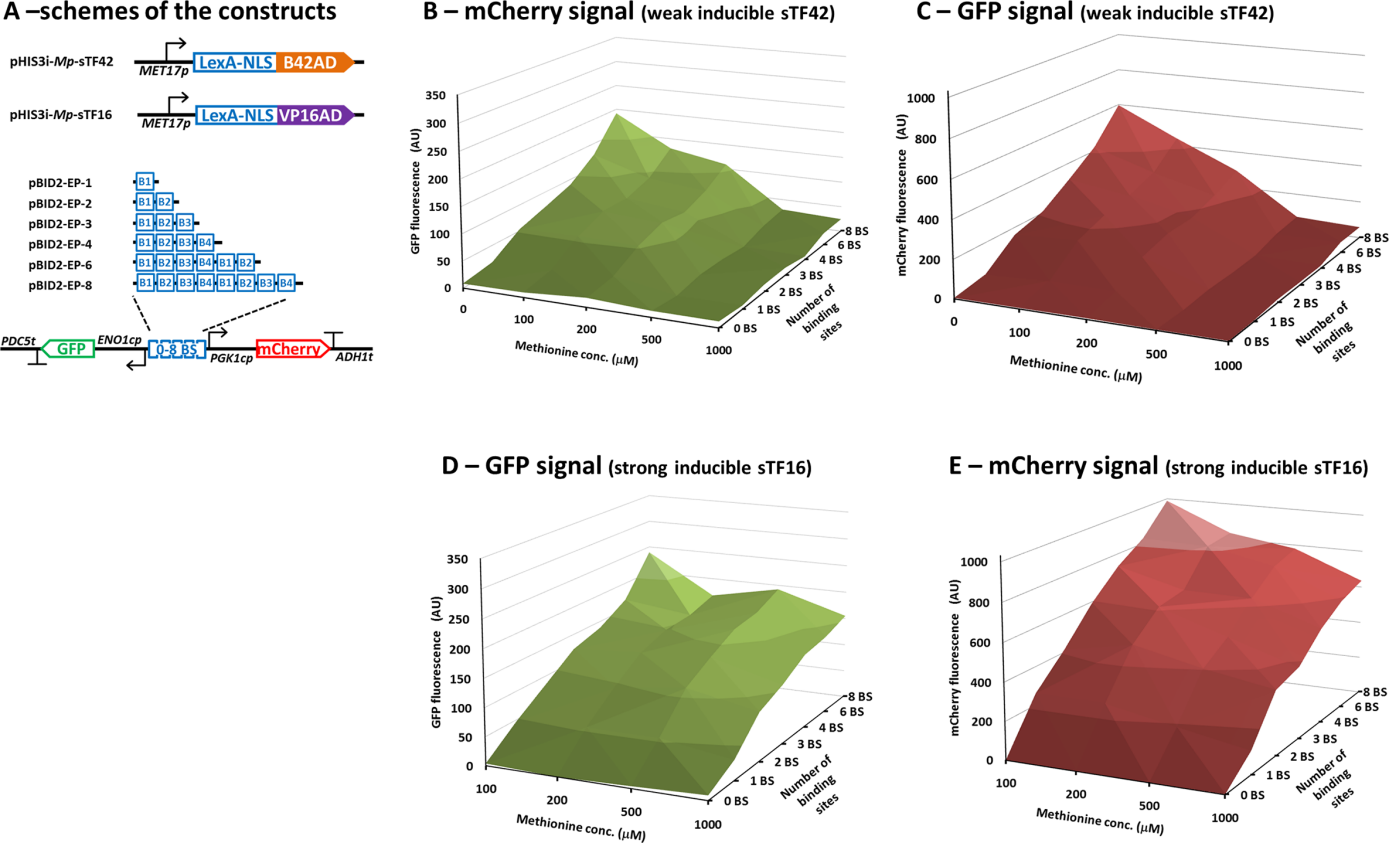
The performance of the inducible synthetic transcription factors. **A)** Schemes of the DNA constructs used in B–E. **B)** Normalised GFP fluorescence of strains with the weak sTF42 and the bidirectional reporter constructs in presence of variable concentrations of methionine. **C)** The normalised mCherry fluorescence of the strains and conditions shown in B. **D)** The normalised GFP fluorescence of the strains with the strong sTF16 and the bidirectional reporter constructs in presence of variable concentrations of methionine. The values in absence of methionine are not shown due to apparent toxicity of the highly expressed sTF16 in this condition resulting in poor growth of the strains and inconsistent fluorescent values. **E)** The normalised mCherry fluorescence of the strains and conditions shown in D. The values represent the averages from at least 3 independent experiments; the standard deviations are not shown for clarity of presentation.

**Fig 6:**
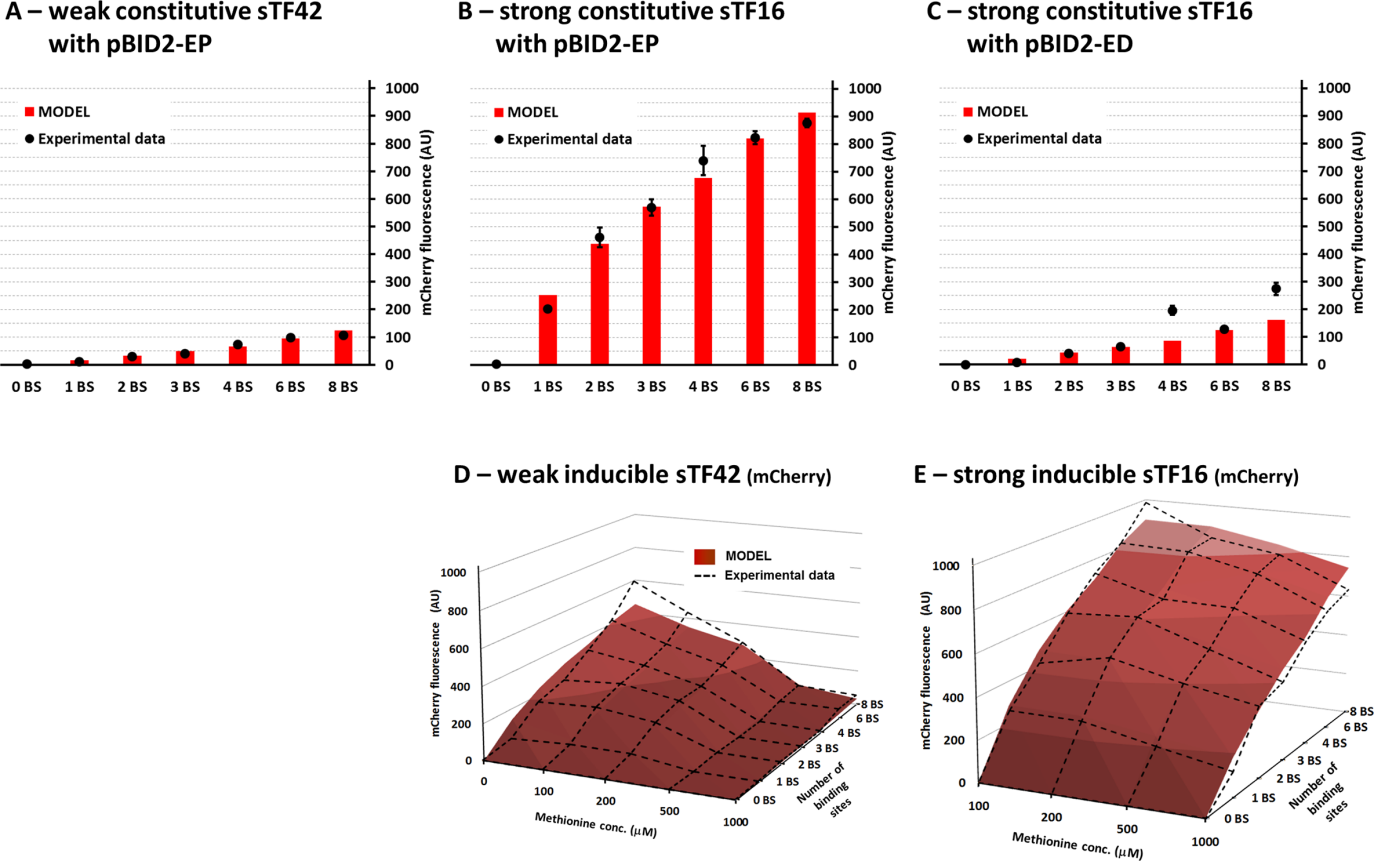
*In silico* modeling of the mCherry fluorescence in selected systems. **A**-**C)** Model predictions for the systems with constitutive sTFs. The red bars correspond to the values predicted by the model while the experimetal values are illustrated by the dots ±standard deviation. In all 3 cases the models corresponding to the systems with 1, 2, and 6 BS were used for parameter estimation while all the other systems (0, 3, 4, and 8 BS) were used for model validation. For the 2 constitutive models with the pBID2-EP (*PGK1*cp for mCherry) (**A** and **B**) we estimated only the kinetic parameter associated to the sTF transcription; all the other parameter values were taken from the model associated to the methionine-induced systems (D and E). For the model associated to the strong constitutive sTF16 system using the pBID2-ED (*DAN1*cp for mCherry) (**C**) we only needed to estimate the kinetic values associated to the recruitment of polymerase associated with sTF to the core promoter; all the other values were taken from the previous model. **D**-**E**) Model predictions for the systems with inducible sTFs. The red surface corresponds to the values predicted by the model while the experimetal values are illustrated by the dashed line.

To generate additional flexibility and versatility into the system we established an alternative module for expression output tuning. This was realized by modulating the expression levels of the synthetic transcription factors. To achieve this, expression of sTF42 and sTF16 was placed under the control of the *MET17* promoter. Combinations of the single genome-integrated cassettes containing either sTF42 or sTF16 with the pBID2-EP set (Fig 5A) were tested at different levels of externally added methionine. The system with the weak sTF42 showed a large dynamic range of the expression outputs ranging from nearly complete down-regulation of the expression in conditions with high concentrations of methionine, to high expression levels in condition without methionine (Fig 5B and 5C). The maximal level of the expression output significantly exceeded the levels observed in the system where the sTF42 was expressed from the *TDH3* core promoter (Fig 3B). However, when the system with the strong sTF16 was tested, the dynamic range was greatly diminished and only minor influence of the methionine concentration was observed (Fig 5D and 5E). The maximal level of expression in this experimental setup was similar to the levels observed in the system where the sTF16 was expressed from the *TDH3* core promoter (Fig 3C). In line with our earlier results for the effect of strong expression of VP16 domain, in the absence of methionine (high induction), cell growth was seriously compromised and consistent results were not obtained for the fluorescence measurements. We did not observe any clear toxic effect of the sTF16 expression in our standard set-up when using 100 μM or higher methionine concentration. However, in order to analyse possible toxicity more closely the growth of yeast cells containing versions of the sTFs was measured with an automated turbidometric analyser (Bioscreen) (Figure F in S1 File). Regardless of concentration (100–1000 μM tested), only the inducible sTF16 showed a growth defect in the absence of methionine and a mild growth phenotype in the presence of methionine. We also assessed the amplification gain in the inducible system in a similar manner as in the constitutive system. The input signal was analysed by expressing the mCherry gene from the *MET17* promoter in a strain cultivated in presence of different concentrations of methionine. These values were compared to the fluorescence levels obtained in the analysis of the inducible systems (Figure D in S1 File). The highest amplification gain was detected in the expression system composed of the sTF16 and the pBID-EP-8 in presence of 500 μM methionine, and it was approximately 10×. The modulation effects of the methionine-induced sTF on the fluorescence levels were well captured by the mathematical model (Fig 6D and 6E) both for the weak sTF42 as well as for the strong sTF16. Collectively, these results establish a modular, bidirectional gene expression system that enables regulation of target gene expression levels over a broad range.

## Discussion

We have developed an orthogonal expression system for *S*. *cerevisiae* consisting of modular DNA parts that can be assembled to functional genetic devises providing precise and predictable control for the expression of single as well as multiple genes. The expression level can be tuned by the choice of the individual modules to provide a gradual range of expression outputs covering the complete spectrum of expression from zero to the level observed for the *TDH3* gene expression, which is considered to be one of the most strongly expressed genes in yeast [35]. An advantage of this system over the previously engineered orthogonal systems is the possibility to use it independent of externally added compounds, such as metabolic inducers, or chemical analogues of tetracycline and estradiol. Therefore, the system should be suitable also in industrial applications which, due to increased cost, generally disfavour the use of additional compounds in the bioprocesses. Another important aspect of the system is the possibility to simultaneously control the expression of two genes from a bi-directional promoter for even highly dissimilar, yet predictable expression levels. This feature can be utilised for constructing metabolic or signalling pathways where different levels of individual proteins are often necessary for optimal functionality (flux or signal transduction). This feature also reduces the number of DNA cassettes that need to be generated to cover a pathway and the genetic loci needed for pathway gene integration. Such a feature can be of use e.g. in industrial strains where known functionally neutral loci within the genome may not be abundant.

The system functions as a fixed-gain amplifier where the input signal, controlling the expression of the sTF, is transformed via the 3 modules (AD, BS, and CP) into the output signal defining the level of expression of the target gene (Fig 1). The fold-change difference between the output and input signals can be seen as an amplification gain of the expression system. The amplification gain in the constitutive system was calculated from results obtained by two different experimental approaches: ii) by comparing the amount of fluorescence of the strains expressing the GFP or mCherry from the input promoter alone (*TDH3*cp) with the level of fluorescence in the strains carrying the complete expression systems (Figure D in S1 File). The amplification gain ranged from 0.1× to around 40× depending on the combination of the modules used for each individual expression system. ii) The system conferring the highest amplification gain (*TDH3*cp–sTF16 + pBID2-EP-8) was also subjected to transcription analysis, where the amplification gain was estimated to be 73× ± 17. An apparent difference of the values obtained from the two approaches could be accounted for a number of reasons associated with the methods, which can influence the measurement and subsequent calculation. For instance, close-to-background fluorescence levels of the input promoter controls is prone to a large error, or the RT-PCR efficiency for different templates might not be identical. The amplification gain in the inducible system was estimated only from fluorescent measurements, and the highest value was around 10× (Figure D in S1 File). This seems to be due to a generally higher level of input signal provided by the *MET17* promoter and close-to-saturation of output signal, which might be a consequence of maximal transcription activity of the chosen BS and CP-modules combination (8BS-*PGK1*cp) (Figure D in S1 File). The previously established gain-tunable genetic amplifiers in *E*. *coli* [8] showed maximal amplification gain of 21×, which is in a similar range as the gains of the systems observed here.

The system can also be seen as a synthetic orthogonal regulon, where the sTF controls the expression of set of genes containing the sTF-dependent promoters of specific strengths. It should be possible to implement additional control circuits regulating the level of the sTF to achieve simultaneous modulation of the whole regulon expression. Thus, the system should be suitable for tuning the flux through a native or heterologous pathway by generating a library of sTF-dependent promoters controlling underlying genes. In the case of modifying endogenous metabolic pathways the broad dynamic range provided by the system should enable balancing of the expression levels of the pathway components and improved flux towards desired products.

The BS module was designed to contain different versions of LexA binding sites. This feature makes these DNA parts suitable for the use of Gibson assembly technique, which is sensitive for repetitive sequences in the DNA regions adjacent to the assembly site. In addition, the heterogeneity of the LexA binding sites should also reduce the possibility for homologous recombination in the repetitive region possibly resulting in instability of the constructs. The modified sites were based on the native LexA binding sites. In case of B1 and B2 on the lexAo1 and lexAo2 present in the regulatory region of the *lexA* gene [32], and in case of B3 and B4 on the *ColE*I operator which contains two partially overlapping LexA binding sites [33]. The modifications introduced to B1, B2, and B4 (B3 is identical to a native site present in the *ColE*I operator; Figure A in S1 File) are in positions that have not been reported to be critical for LexA binding. Furthermore, the same bases are also present in other native LexA binding sites [32]. Qualitative *in vitro* analysis of LexA binding to B1-B4 performed by EMSA (Figure C in S1 File) showed equivalent functionality of the binding sites. The conditions in the EMSA resembled closely the conditions used in previous reports that focused on detailed analysis of the LexA binding properties [36]. However, we cannot draw any conclusions about the LexA binding affinities and we cannot exclude the possibility that the modifications introduced affected the binding affinity. The predictable behaviour of the modified LexA binding sites was, however, observed in the experiments where the increasing number of the BSs (1–4) resulted in a consistent gradual increase of the activity of the corresponding synthetic promoters (Figs 3–5) and therefore rendering functional BS modules.

The core promoters (CP module) were initially selected from the promoters of highly expressed genes, commonly used for heterologous gene expression in yeast [37,38]. This was motivated by the assumption that the core part of these promoters is responsible for efficient recruitment of the general transcription machinery. The differences in the native promoter strengths was expected to be caused by the specific transcription activators whose binding sites are positioned upstream of the generic core sequence. There are numerous examples of sites which are essential and in many cases also sufficient for a promoters’ activity; the Gcr1 sites in the *PDC1*, *TPI1*, *TDH3*, *ENO1*, and *PGK1* promoters located in regions typically -350 to -600 relative to the translation start [39,40]; the Rap1 sites in the *PDC1*, *TDH3*, *ENO1*, and *PGK1* promoters in the similar regions [39,41]; and also the Gal4 sites in the *GAL1* promoter located in a region -330 to -450 relative to the translation start [31,42]. Optimally, for predictable behaviour, the selected and engineered core promoter elements should provide a constant expression output irrespective to different growth conditions. Indeed, the *THD3*cp activity seems to be unaffected by the carbon source and the growth status of the cells. The expression of the sTF16 gene under *TDH3*cp and the mCherry reporter gene under sTF-dependent promoter, as determined by the RT-PCR analysis, conferred very stable and similar expression patterns during 24 hour cultivation in defined (SC) as well as complex (YP) media in presence of either glucose or ethanol (Figure E in S1 File). However, a minor decline in the expression of both sTF and mCherry was observed in cells during exponential growth on glucose (particularly in YPD). This could be a result of the RT-PCR signal normalization, which is especially challenging when monitoring diverse conditions in one experiment. This is due to a lack of known control genes conferring absolutely stable expression. We used the *UBC6* gene as a normalization control, which has been shown to be stably expressed across a large spectrum of conditions [43]. Therefore, we cannot completely exclude the possibility that the *TDH3*cp might undergo minor transcriptional regulation due to growth conditions.

An important aspect in the characterization of introduced expression system is the assessment of its influence on the host. In an ideal case, there should be no interactions other than those intended by the design or purpose of the expression system. Often, however, heterologous DNA parts cause unwanted changes into host organism’s regulatory or metabolic networks and sometimes this results in toxicity. A known example, relevant to this work, is squelching caused by high expression levels of heterologous transcription factors containing the VP16 activation domain [44]. Squelching, or transcriptional interference, is a process in which the introduced transcription factor competes for activating factors with the native transcription factors that in turn may result in insufficient expression of essential genes. In strains expressing sTF16 from the *MET17* promoter, we observed a strong growth defect in absence of methionine, and a mild growth phenotype was detected in detailed growth assays in the presence of 100–1000 μM of methionine. Identical cell growth compared to a control strain was observed for the strain expressing the sTF16 from *TDH3*cp (Figure F in S1 File). The expression level achieved by the *TDH3*cp was lower than the expression levels provided by the *MET17* promoter in any tested methionine concentration (Figure D in S1 File). Together these results led us to a hypothesis that (at least) two thresholds can be identified for the levels of the sTF16 associated with its toxicity: i) The level achieved by the *TDH3*cp, up to which the sTF16 is harmless for the cells; and ii) the level achieved by fully active *MET17* promoter, which results in severe toxicity. The levels between these two thresholds seem to have only minor, negative consequences for the host cell.

The mathematical model developed in this paper for the expression system captures the combinatorial effect of the above mentioned modules (AD, BS, and CP) and illustrated the diverse expression output as monitored by the fluorescence levels in the experiments. In particular, for the constitutive systems the models showed the increase in the fluorescence levels depending on the number of sTF-specific binding sites following closely the experimental measurements. Also for the methionine-induced systems, the associated models successfully captured the two very different dynamics given by the use of either the weak sTF42 or the strong sTF16. The model’s parameters were fitted using only a fraction of the experimental data sets while the rest of the data was used to test the predictive power of the model. However, the numerical setup (obtained through parameter estimation) that leads to a good model fit is in general not unique [45,46]. This, in turn, leads to a sensitivity of the model analysis to the choice of the numerical values for the kinetic parameters.

Other expression systems developed so far for *S*. *cerevisiae* offer a spectrum of features partially overlapping with our system. Collectively, these systems form a solid basis for engineering of robust, well-defined, and predictable expression systems for this industrially relevant production host. In an elegant study of Ito and co-workers [20] an expression system was developed which shares a number of similarities to the system presented here. The system is based on the LexA sTF and the authors showed importance of the binding sites and core promoter choices for the performance of the synthetic promoter. In addition, the choice of terminator seemed to have a significant effect on the level of expressed heterologous gene, and therefore it can be considered as an additional module in controlling the output of the system. In line with our findings, the authors also tested different levels of the sTF expression and observed only minimal influence on system’s performance. In the present study, we, on the other hand, show that the use of different activation domains in the sTF could be efficiently employed for modulation of the system’s output. Also the use of bidirectional synthetic promoters represents a significant improvement for the future applicability of the expression system. Furthermore, our study characterizes modified LexA-binding sites having beneficial properties (improved binding, avoidance of repetitiveness) that should prove useful in future studies. An important study by Ottoz and co-workers [18] showed tight dependences of expression strength with the transcription factor binding sites in the synthetic promoter and the choice of the activation domain in the sTF. These features were all supported by the associated mathematical model. Their system was, however, based on β-estradiol-activated sTFs which might cause limitations for industrial use. We were able to demonstrate that the same range of expression levels can be achieved by an external-compound-independent system. Another recent study [17] demonstrated the importance of the core promoter modules in the precise regulation of synthetic expression system. Based on that study, we introduced the *DAN1* core promoter into our system (Fig 3B). Interestingly, some of the versions of the expression system containing the *DAN1*cp but no other core promoter (pBID2-ED-4 and pBID2-ED-8) displayed an unexpectedly high expression output. The B3 and B4, present in these constructs, are derived from the *ColEI* operator and they retained the partially overlapping organization, which has been shown to cause not only bending of the DNA bound by the LexA protein but also cooperativity of the binding [47]. It is possible that, these phenomena in combination with the positioning of the *DAN1*cp in these constructs can have caused more favourable assembly of the transcription machinery resulting in higher expression of the reporter gene. When the spacing of the B3-B4 and the *DAN1*cp was different (such as in pBID2-ED-6), or when another core promoter was tested, no such influence for the expression was observed. This illustrates some of the challenges we are still facing in the development of robust synthetic expression systems consisting of DNA modules.

The system described in this study expands the family of existing gene expression regulation systems by providing a compact platform for simultaneous expression of multiple genes in *S*. *cerevisiae*.

## Supporting Information

S1 File **Figure A in S1 File**: DNA sequences used for EMSA and the LexA binding sites. **Figure B in S1 File**: Sequences of the core promoters used in the study. **Figure C in S1 File**: *In vitro* binding of the LexA binding sites to the purified sTF16 (EMSA). **Figure D in S1 File**: The assessment of the amplification gain in the expression systems. **Figure E in S1 File**: Transcription analysis of the constitutive system based on strong sTF16 and pBID2-EP-8, an assessment of the system**’**s performance in different growth conditions. **Figure F in S1 File**: The assessment of toxicity of the selected DNA constructs in different concentrations of methionine. **Figure G in S1 File**: *In silico* modeling of the system with strong constitutive sTF16 and pBID2-ED. **Figure H in S1 File**: The residuals distributions for the considered systems. **Table A in S1 File**: List of plasmids. **Table B in S1 File**: List of primers. **Table C in S1 File**: The biochemical reaction network for the methionine induced sTF system. **Table D in S1 File**: The biochemical reaction network for the constitutive sTF system. **Table E in S1 File**: Cellular localization of the components included in the mathematical model. **Table F in S1 File**: Model parameters values. **Table G in S1 File**: *R*^2^values measuring the goodness of fit between model predictions and experimental measurements
(DOCX)
