# Supplementary material for: Synthetic Transcription Amplifier System for Orthogonal Control of Gene Expression in Saccharomyces cerevisiae
Source: PLoS One. 2016 Feb 22;11(2):e0148320. doi: 10.1371/journal.pone.0148320 (PMC4762949; doi:10.1371/journal.pone.0148320)
Supplement: S1 File — Figure A in S1 File: DNA sequences used for EMSA and the LexA binding sites. Figure B in S1 File: Sequences of the core promoters used in the study. Figure C in S1 File: In vitro binding of the LexA binding sites to the purified sTF16 (EMSA). Figure D in S1 File: The assessment of the amplification gain in the expression systems. Figure E in S1 File: Transcription analysis of the constitutive system based on strong sTF16 and pBID2-EP-8, an assessment of the system’s performance in different growth conditions. Figure F in S1 File: The assessment of toxicity of the selected DNA constructs in different concentrations of methionine. Figure G in S1 File: In silico modeling of the system with strong constitutive sTF16 and pBID2-ED. Figure H in S1 File: The residuals distributions for the considered systems. Table A in S1 File: List of plasmids. Table B in S1 File: List of primers. Table C in S1 File: The biochemical reaction network for the methionine induced sTF system. Table D in S1 File: The biochemical reaction network for the constitutive sTF system. Table E in S1 File: Cellular localization of the components included in the mathematical model. Table F in S1 File: Model parameters values. Table G in S1 File: R2values measuring the goodness of fit between model predictions and experimental measurements (DOCX) [file pone.0148320.s001.docx]

# Supplementary information

# Synthetic transcription amplifier system for orthogonal control of gene expression in *Saccharomyces cerevisiae*

Anssi Rantasalo, Elena Czeizler, Riitta Virtanen, Juho Rousu, Harri Lähdesmäki, Merja Penttilä, Jussi Jäntti and Dominik Mojzita

## Supplementary methods

### Construction of the sTF expression constructs

The plasmid pHIS3i-*Mp*-sTF42 is a modified version of p-pHluorin2-LexA-AD [1] where the *Fse*I – *Sal*I fragment was removed and the plasmid re-ligated after Klenow-polymerase treatment. The resulting plasmid contains a fusion gene (LexA DNA-binding domain + SV40-NLS + B42 activation domain; sTF42) under control of the *MET17* promoter, and it has a backbone of pYX022 integrative vector (R&D Systems). The plasmid pHIS3i-*Mp*-sTF16 was constructed from pHIS3i-*Mp*-sTF42 by replacing of the B42 activation domain with VP16 activation domain. The VP16 activation domain was amplified from the plasmid B4872 (VTT collection) with primers 971 and 972, and assembled (Gibson Assembly kit (NEB)) with *Eco*RI – *Sac*I fragment (5749bp) of the pHIS3i-*Mp*-sTF42 plasmid. The plasmids pHIS3i-*Tcp*-sTF42 and pHIS3i-*Tcp*-sTF16 were constructed from the above plasmids by replacing of the *MET17* promoter with the *TDH3* core promoter (*Tcp*). The *TDH3*cp was amplified with primers 1033 and 1034 from the synthetic DNA construct (TDH3cp-TPI1cp (6×BS)) obtained from GeneArt (Life Technologies). The PCR products were assembled with *Sal*I – *Xba*I fragments of the respective plasmids by the Gibson Assembly kit (NEB). The control plasmid pHIS3i-(wop)-sTF16, without the promoter controlling the expression of the sTF, was constructed by self-ligation of the Klenow-polymerase-treated *Sal*I – *Xba*I fragment (5363bp) of the pHIS3i-*Tcp*-sTF16. All the ligation mixes were transformed to *E. coli* (TOP10) by electroporation and all the constructs were confirmed by sequencing.

### Construction of the pBID1 plasmids set

The construction of preliminary plasmid versions was initiated on the plasmid p-pHluorin2-LexA-AD [1]. First, the 8×BS in the original plasmid were replaced by 2×BS with simultaneous removal of the extra GAL1 promoter sequences. This was performed by assembling the *Aat*II – *Nco*I fragment (7332bp) of the p-pHluorin2-LexA-AD plasmid with a PCR product (primers 858 and 861) derived from the same plasmid, by the Gibson Assembly kit (NEB). The resulting plasmid (p-pHluorin2-2×BS) was used as a template for the second step. Here the *Fse*I – *Nco*I fragment (6636bp) of the plasmid was ligated with two PCR products derived from the p-pHluorin-2×BS plasmid; one PCR product (primers 861 and 870) digested with *Xma*I and *Nco*I, and a second PCR product (primers 869 and 871) digested with *Xma*I and *Fse*I. This resulted in a modified p-pHluorin2-2×BS-Sma plasmid with *Sma*I/*Xma*I site in the 5’-end of the 2×BS sequence. Two consecutive ligations were done to create the 6×BS version, first the p-pHluorin2-2×BS-Sma (digested with *Fse*I – *Xma*I) was ligated to *Fse*I – *Xma*I digested PCR product (primers 869 and 872, p-pHluorin-2×BS-Sma as a template) to generate p-pHluorin2-4×BS-Sma; and second, the p-pHluorin2-4×BS-Sma (digested with *Fse*I – *Xma*I) was ligated to *Fse*I – *Xma*I digested PCR product (primers 869 and 872, p-pHluorin-2×BS-Sma as a template) to generate p-pHluorin2-6×BS-Sma. This plasmid was digested with *Sma*I and the linearized DNA was assembled by the Gibson Assembly kit (NEB) with 3 PCR products: 1) *PDC1*cp amplified from *S. cerevisiae* genomic DNA with primers 982 and 893; 2) mCherry ORF amplified from pMAM12 (gift from Michael Knop; Heidelberg) with primers 898 and 899; 3) *PGK1* terminator amplified from *S. cerevisiae* genomic DNA with primers 902 and 903. The resulting plasmid, p-BID-GAL1cp(pHl2)+PDC1cp(mCherry), was further modified by exchanging of the pHluorin2 gene by the GFP gene. The plasmid was digested with *Pac*I and *Sal*I and ligated with two PCR products: 1) *Pac*I digested bidirectional promoter amplified form the p-BID-GAL1p(pHl2)+PDC1p(mCherry) with primers 892 and 901; and 2) *Pac*I – *Sal*I digested GFP-ORF amplified from the pUG36 plasmid [2] with primers 904 and 905. The ligation resulted in plasmids, p-BID-GAL1cp(GFP)+PDC1cp(mCherry).

The construction of the pBID1-PGA-6 was started by transferring of the *Sma*I – *Kpn*I fragment of the synthetic TDH3cp-TPI1cp (6×BS) (for full sequence, see below – Synthetic DNA constructs) construct into *Ecl136*II – *Kpn*I digested pSR406 [3]. The resulting plasmid was digested with *Bam*HI + *Sal*I and the fragment (4893bp) was ligated to the *Bam*HI – *Sal*I fragment (2031bp) of the p-BID-GAL1cp(GFP)+PDC1cp(mCherry), which resulted in the pBID1-PGA-6. The pBID1-PGB-6 plasmid was constructed by *Pac*I digestion of the pBID1-PGA-6 and re-ligation of the bidirectional promoter in opposite orientation. The pBID1-TTA-6 and pBID1-TTB-6 plasmids were constructed by transferring the bidirectional promoter (*Pac*I digestion) from the synthetic DNA construct, TDH3cp-TPI1cp (6×BS), into *Pac*I fragment (6345bp) of the pBID1-PGA-6 plasmid (two possible orientation of the bidirectional promoter resulted in two versions of the plasmid, which is denoted by A or B letter in the plasmids’ name). Analogously, the pBID1-EPA-6 and pBID1-EPB-6 plasmids were constructed by *Pac*I – mediated transfer of the bidirectional promoter from the synthetic DNA construct, ENO1cp-PGK1cp (6×BS), into the *Pac*I fragment (6345bp) of the pBID1-PGA-6 plasmid. All the ligation mixes were transformed to *E. coli* (TOP10) by electroporation and all the constructs were confirmed by sequencing.

The pBID1 versions with the full-length promoters were constructed by the Gibson assembly. The full-length promoters were amplified from *S. cerevisiae* genomic DNA and assembled with the *Pac*I digested (6345bp fragment) plasmid pBID1-PGA-6. The PCR products for the assembly were obtained with primers: 1) the *PDC1* promoter (800bp) primers 1079 and 1081 and the *GAL1* promoter (550bp) primers 1078 and 1080 for the plasmid pBID1-PGA-F; 2) the *PDC1* promoter (800bp) primers 1081 and 1082 and the *GAL1* promoter (550bp) primers 1080 and 1083 for the plasmid pBID1-PGB-F; 3) the *TPI1* promoter (560bp) primers 1084 and 1086 and the *TDH3* promoter (650bp) primers 1085 and 1087 for the plasmid pBID1-TTA-F; 4) the *TPI1* promoter (560bp) primers 1086 and 1089 and the *TDH3* promoter (650bp) primers 1087 and 1088 for the plasmid pBID1-TTB-F; 5) the *ENO1* promoter (1000bp) primers 1093 and 1094 and the *PGK1* promoter (760bp) primers 1092 and 1095 for the plasmid pBID1-EPA-F; 6) the *ENO1* promoter (1000bp) primers 1091 and 1093 and the *PGK1* promoter (760bp) primers 1090 and 1092 for the plasmid pBID1-EPB-F. All the ligation mixes were transformed to *E. coli* (TOP10) by electroporation and all the constructs were confirmed by sequencing.

### Construction of the pBID2 plasmids set

The starting material for the construction of pBID2-EP-1-4 was the plasmid pBID1-EPA-6. The PCR template for 1-3×BS versions was a *Hind*III - *Sph*I fragment (1117bp) of the plasmid pBID1-EPA-6. Three individual PCR reactions were run for 1-3×BS-containing fragments: 1) 1×BS-*PGK1*cp-mCherry with primers 1009 and 1010; 2) 2×BS-*PGK1*cp-mCherry with primers 1009 and 1011; and 3) 3×BS-*PGK1*cp-mCherry with primers 1009 and 1012. The plasmid pBID1-EPA-6 as well as the three PCR products were digested with *Spe*I and *Bam*HI (removing the 6×BS-*PGK1*cp-mCherry part of the pBID1-EPA-6 plasmid) and the corresponding DNA fragments were ligated to form pBID2-EP-1-3. The 4×BS version was constructed by self-annealing of the primers 1013 and 1014 followed by filling of the ssDNA ends by DNA polymerase (Phusion, Life Technologies). The resulting product was assembled with *Spe*I – *Hind*III fragment (6741bp) of the pBID1-EPA-6 by the Gibson Assembly kit (NEB). The pBID2-EP-0 was constructed by self-ligation of the *Spe*I – *Nhe*I digested pBID2-EP-1. The 6- and 8×BS versions were constructed by ligating the *Nhe*I – *Bam*HI fragment (5905bp) of the pBID2-EP-4 with 1) *Spe*I – *Bam*HI fragment (966bp) of the pBID2-EP-2 – for pBID2-EP-6, and 2) *Spe*I – *Bam*HI fragment (1005bp) of the pBID2-EP-4 – for pBID2-EP-8. All the ligation mixes were transformed to *E. coli* (TOP10) by electroporation and all the constructs were confirmed by sequencing.

The pBID2-ED-6 and the pBID2-E156-6 plasmids were constructed by the Gibson assembly. The *DAN1* and the *YLR156W1* core promoters were amplified from *S. cerevisiae* genomic DNA with primers 1126+1129 and 1141+1144, respectively. The mCherry was amplified from the pBID1-PGA-6 plasmid with primers 1123+1124. The corresponding DNA fragments were assembled with the *Nhe*I – *Bam*HI digested (5950bp fragment) plasmid pBID1-PGA-6. The remaining plasmids containing the *DAN1* and the *YLR156W1* core promoters were constructed by the replacement of *Kpn*I – *Nhe*I regions of pBID2-ED-6 and pBID2-E156-6 by the *Kpn*I – *Nhe*I regions of pBID1-EP-1, pBID1-EP-2, pBID1-EP-3, pBID1-EP-4, and pBID1-EP-8, respectively. This was achieved by classical cloning. The pBID2-ED-0 and the pBID2-E156-0 plasmids were constructed by *Nhe*I – *Spe*I digestions of the corresponding 6×BS versions followed by the re-circularization. All the ligation mixes were transformed to *E. coli* (TOP10) by electroporation and all the constructs were confirmed by sequencing.

### Construction of the pHIS3i-*Mp*-mCh plasmid

The plasmid pHIS3i-*Mp*-mCh was constructed from the pHIS3i-*Mp*-sTF42 plasmid by replacement of the sTF42 gene by the mCherry gene. This was done by blunt-end ligation of the Klenow filled-in *XbaI-NheI* fragment of pHIS3i-*Mp*-sTF42 (5132 bp) into the Klenow filled-in *PacI-SapI* fragment of pBID2-EP-8 (1307 bp). The ligation mix was transformed to *E. coli* (TOP10) by electroporation and the resulting pHIS3i-*Mp*-mCh plasmid was confirmed by sequencing.

### Synthetic DNA constructs

TDH3cp-TPI1cp (6×BS)

*CCCGGG*TTGTCCTCTGAGGACATAAAATACACACCGAGATTCATCAACTCATTGCTGGAGTTAGCATATCTACAATTGGGTGAAATGGGGAGCGATTTGCAGGCATTTGCTCGGCATGCCGGTAGAGGTGTGGTCAATAAGAGCGACCTCATGCTATACCTGAGAAAGCAACCTGACCTACAGGAAAGAGTTACTCAAGAATAAGAATTTTCGTTTTAAAACCTAAGAGTCACTTTAAAATTTGTATACACTTATTTTTTTTATAACTTATTTAATAATAAAAATCATAAATCATAAGAAATTCGC*GGATCCTTTAATTAA*TTATGTGTGTTTATTCGAAACTAAGTTCTTGGTGTTTTAAAACTAAAAAAAAGACTAACTATAAAAGTAGAATTTAAGAAGTTTAAGAAATAGATTTACAGAATTACAATCAATACCTACCGTCTTTATATACTTATTAGTCAAGTAGGGGAATAATTTCAGGGAACTGGTTTCAACCTTTTTTTTCAGCT*ACTAGT*TCAAATGAGCTAGGGACCTGG**CTGTATATAAACAC**AGCATAA**CTGTATATATACCC**AGGGGACCTGG**CTGTATATAAACAC**AGCATAA**CTGTATATATACCC**AGGGGACCTGG**CTGTATATAAACAC**AGCATAA**CTGTATATATACCC**AGGGATGATAATGCGATT*AAGCTT*TACCTTTGGCTCGGCTGCTGTAACAGGGAATATAAAGGGCAGCATAATTTAGGAGTTTAGTGAACTTGCAACATTTACTATTTTCCCTTCTTACGTAAATATTTTTCTTTTTAATTCTAAATCAATCTTTTTCAATTTTTTGTTTGTATTCTTTTCTTGCTTAAATCTATAACTACAAAAAACACATACAT*TTAATTAAAGTCGAC*GCTAATTAACATAAAACTCATGATTCAACGTTTGTGTATTTTTTTACTTTTGAAGGTTATAGATGTTTAGGTAAATAATTGGCATAGATATAGTTTTAGTATAATAAATTTCTGATTTGGTTTAAAATATCAACTATTTTTTTTCACATATGTTCTTGTAATTACTTTTCTGTCCTGTCTTCCAGGTTAAAGATTAGCTTCTAATATTTTAGGTGGTTTATTATTTAATTTTATGCTGATTAATTTATTTACTTTCGTATTCGGTTTTGTACCTTTAGCTATGATCTTAGCTAATTGAA*GGTACC*

ENO1cp-PGK1cp (6×BS)

*CCCGGG*AAGCAATGGATTATGCCATAAGTATTGAAAATATTGTGTAATATTTGTATATATAATATGTGCACAAAATTACTACAAAATAAACAACTTTTTCTCTCCCGGAGAACAAAGTAAGTGGAAAAAATTATACTAATAACAATCTAAAAATGTTTATTAGCTAGGATTATACACATAAATATATATATATGTGTGTGTCTATATTTACATATACTAGACCGCAAAAAAAACATAAGGGCATCACTCATAAGAATAATAATATTAAGGGAGGGAAAAACACATTTATATTTCATTACATTTTTT*GGATCCTTTAATTAA*GTGTTTGTGTGTTGATAAGCAGTTGCTTGGTTTTTTATGAAAAATAGCTAGAAGGAATAAGGGATTACAAGAGAGATGTTACAAGAAAGAAGTAAAATAAATTTGATTAATATTGCCATTATCAAAAGCTATTTATATGTTGAAATCGTGGAGATCATGTGTGCCAGAAAAGGCCACAGTTTCCGGGGAGA*ACTAGT*TCAAATGAGCTAGGGACCTGG**CTGTATATAAACAC**AGCATAA**CTGTATATATACCC**AGGGGACCTGG**CTGTATATAAACAC**AGCATAA**CTGTATATATACCC**AGGGGACCTGG**CTGTATATAAACAC**AGCATAA**CTGTATATATACCC**AGGGATGATAATGCGATT*AAGCTT*AAGGGGGTGGTTTAGTTTAGTAGAACCTCGTGAAACTTACATTTACATATATATAAACTTGCATAAATTGGTCAATGCAAGAAATACATATTTGGTCTTTTCTAATTCGTAGTTTTTCAAGTTCTTAGATGCTTTCTTTTTCTCTTTTTTACAGATCATCAAGGAAGTAATTATCTACTTTTTACAACAAA*TTAATTAAAGTCGAC*GCGATTTAATCTCTAATTATTAGTTAAAGTTTTATAAGCATTTTTATGTAACGAAAAATAAATTGGTTCATATTATTACTGCACTGTCACTTACCATGGAAAGACCAGACAAGAAGTTGCCGACAGTCTGTTGAATTGGCCTGGTTAGGCTTAAGTCTGGGTCCGCTTCTTTACAAATTTGGAGAATTTCTCTTAAACGATATGTATATTCTTTTCGTTGGAAAAGATGTCTTCCAAAAAAAAAACCGATGAATTAGTGGAACCAAGGAAAAAAAAAGAGGTATCCTTGATTAAGGAACA*GGTACC*

### Mathematical modelling

We developed 4 mechanistic dynamic models in terms of ordinary differential equations corresponding to the methionine induced sTF and the constitutive sTF systems using either sTF16 or sTF42 constructs. The only difference between the models corresponding to the constitutive and the induced systems stands in the sTF transcription process illustrated by reaction 2 from the S4 Table and reactions 2-4 from the S3 Table, respectively. The only difference between the models for the systems using the sTF16 or sTF42 constructs stands in the kinetic rates associated to 2 reactions: i) the association of the polymerase with the sTF’s bound to their specific DNA sites and ii) the degradation rate of the sTF proteins corresponding to the two transcription factors sTF16 or sTF42. The model associated to the methionine induced systems consists of 16 reactions involving 18 species and 20 kinetic parameters. The model associated to the constitutive systems consists of 14 reactions involving 13 species and 18 kinetic parameters. In each of these 4 models we also need to specify the number of sTF-specific binding sites, which varied in the experiments between 0 and 8. Starting from the model associated to the constitutive systems using the sTF16 construct we derived an additional 5^th^ model corresponding to the case when the mCherry core promoter is switched from pBID2-EP to pBID2-ED. The only difference between these two last models stands in the kinetic rates for the association of polymerase bound to sTF and the core promoter. The SBML source codes of these 5 models were deposited in the BioModels database [4] and assigned the identifiers MODEL1510230001, MODEL1510230002, MODEL1510230003, MODEL1510230004, and MODEL1510230005.

Since the 5 considered models have many parts in common, our goal was to identify a common set of values for the kinetic parameters that would explain all the observed experimental data. This is known as parameter estimation or inverse modelling, a problem well-known to be computationally challenging for complex systems (see e.g., [5]). We performed this parameter estimation task in 3 steps as described below.

Step 1: Identification of parameter values for the methionine induced models. We divided the 63 experimental measurements into a training set and a validation set. The training set consisted of (i) the sTF16 constructs with 0, 1, 2 and 6 sTF-binding sites from 100 and 200 µM methionine levels, (ii) the sTF16 constructs with 2 and 6 sTF-binding sites from 500 and 1000 µM methionine levels, and (iii) the sTF42 constructs with 1 and 6 sTF-binding sites from 0, 100, 200, 500 and 1000 µM methionine levels. All the other 41 experimental measurements formed the validation set. The mathematical model used for parameter estimation was obtained by putting together the 22 models corresponding to the chosen experimental setups and summed up to 352 reactions involving 396 species. However, the number of kinetic parameters remained 20 since all models had the same kinetic rates for the corresponding reactions. During this phase we also estimated the quantity of methionine still remaining in the system at the 16h time point when the experimental measurements were done (since the model does not include any reactions about the methionine transport into the nucleus or its consumption/production within the cell). For parameter estimation we used simulated annealing [6] as a global optimization method to minimize the sum of squares objective function

$$f\left( X,\theta\right)=\sum_{n} {(Y_{n}-X_{n}\left( \theta\right))}^{2}$$

where $Y_{n}$ and $X_{n}\left( \theta\right)$ stand for the n-th experimental measurement and the model prediction for the n-th data point, respectively, and $\theta$ denotes the model parameters. All model analysis and simulations were done by using the software COPASI [7].

The identified parameter values were then used for model validation over the remaining 41 experimental measurements. Figure H^D-E^ illustrates the model predictions for all 63 experimental instances (35 for the systems using the sTF42 constructs and 28 for the systems using the sTF16 constructs). We plotted in the same figure both the numbers obtained during the parameters estimation phase and those from the model validation phase so that we could overlay the model predictions on the experimental measurements (Figure H^D-E^).

Step 2: Identification of parameter values for the constitutive models. Since the only difference between the models corresponding to the constitutive and the induced systems stands in the sTF transcription process, we could transfer in the constitutive model the values for all kinetic parameter except for the sTF transcription rate. To estimate this parameter, we partitioned the experimental data for the constitutive systems into a training set consisting of the constructs with 1, 2 and 6 sTF-binding sites for both sTF16 and sTF42 systems and a validation set consisting of all the other 8 data sets, see Figure H^A-B^. The mathematical model used during the parameter estimation phase consisted of 84 reactions involving 78 species and 18 kinetic parameters (out of which only 1 needed to be estimated).

Step 3: Identification of parameter values for the model associated to the systems using the sTF16 and the pBID2-ED (instead of the previously used pBID2-EP). In this new model, we only needed to re-estimate the kinetic parameters corresponding to the the association of polymerase bound to sTF with the core promoter (reaction 7 in S4 Table), all the other reactions having the same kinetics as in the previous model. The training set used for this parameter estimation consisted of the sTF16 constructs with 1, 2, 6 sTF-binding sites; the other 4 data sets corresponding to the systems with 0,3,4 and 8 sTF-binding sites forming the validation set, see Figure H^C^. The mathematical model used during the parameter estimation phase consisted of 42 reactions involving 42 species and 18 kinetic parameters (out of which only 2 needed to be estimated).

The model under-estimation of the fluorescence levels for the cases having 4 and 8 sTF- binding sites when using the pBID2-ED could indicate that there is an additional association effect between the actual binding sites used in the BS module and the core promoter; these two cases are the only ones having B4 immediately preceding the *DAN1*cp . We tested *in silico* this hypothesis by allowing the models corresponding to these 2 constructs to have different kinetic parameters for the association of polymerase bound to sTF and the core promoter than the other 5 models corresponding to the constructs with 0, 1, 2, 3 and 6 binding sites (Figure G). For this we used as training data the measurements for 1, 4, 6 and 8 binding sites and as validation set the ones for the 0, 2 and 3 binding sites. The mathematical model used during this parameter estimation phase consisted of 56 reactions involving 56 species and 18 kinetic parameters (out of which we estimated 4). The model predictions obtained when using this hypothesis improved significantly as illustrated in the Figure G.

In order to assess the goodness-of-fit between the model predictions and the data we computed the $R^{2}$score, also known as the coefficient of determination, which measures how well the model fit succeeds in explaining the data variation:

$$R^{2}=1-\frac{{SS}_{res}}{{SS}_{tot}}$$

where ${SS}_{res}=\sum_{i=1}^{n} {(Y_{i}-X_{i}(\theta))}^{2}$ and ${SS}_{tot}=\sum_{i=1}^{n} {(Y_{i}-\bar{Y})}^{2}$ for $\bar{Y}=\frac{1}{n}\sum_{i=1}^{n} Y_{i}$. While the $R^{2}$ values are always between 0 and 1, in general the better the fit is the closer to 1 this value is, since a fit is considered to be good if the distances between the experimental measurements and the model predictions are small and unbiased. The computed $R^{2}$ values for all models were included in S6 Table indicating a very good fit of the data fluctuations especially for the systems using the strong sTF16 (both constitutive and methionine-induced) as well as for the constitutive weak sTF42. The worst score is obtained for the methionine-induced system using the weak sTF42 for the 1000 µM methionine level, but this seems to be caused more by measurements difficulties due to the small numbers (for the values corresponding to the cases with 3 and 4 sTF-specific binding sites). Since the $R^{2}$ values alone are not always enough to determine whether the model predictions are biased, we also assessed the residual plots for all these systems (Figure H). A good model fit should generate residuals that are equally distributed along the X-axis, normally distributed around 0 and without showing systematic trends in their distribution [8,9]. The residual distributions for the models associated to the systems using the strong sTF16 (both constitutive and methionine-induced) as well as for the constitutive weak sTF42 re-confirm a very good quality fit for these cases (Figure H^A-F^). Although the $R^{2}$ scores for the methionine-induced weak sTF42 are also high (except the one for the 1000 µM methionine level), the associated residual distributions indicate slight biases of the model towards overestimating/underestimating some of the experimental measurements.

## Figure A: DNA sequences used for EMSA and the LexA binding sites

A – Cy-5-labelled DNA fragments containing individual LexA-binding sites

B – Unlabelled DNA competitor containing 4 LexA BSs corresponding to the sequence present in pBID2-EP-4

C – Sequence of the LexA binding sites in the regulatory region of the *lexA* gene [10]. These BSs, with modified bases highlighted in red, were used in this work as B1 and B2 versions of the LexA binding sites.

D – Sequence of 2 LexA binding sites in the pSH18-34 plasmid [11], where this sequence (*ColEI* operator) is used in four consecutive repeats forming 8 BS for LexA. These BSs, with the modified base highlighted in red, were used in this work as B3 and B4 versions of the LexA binding sites.


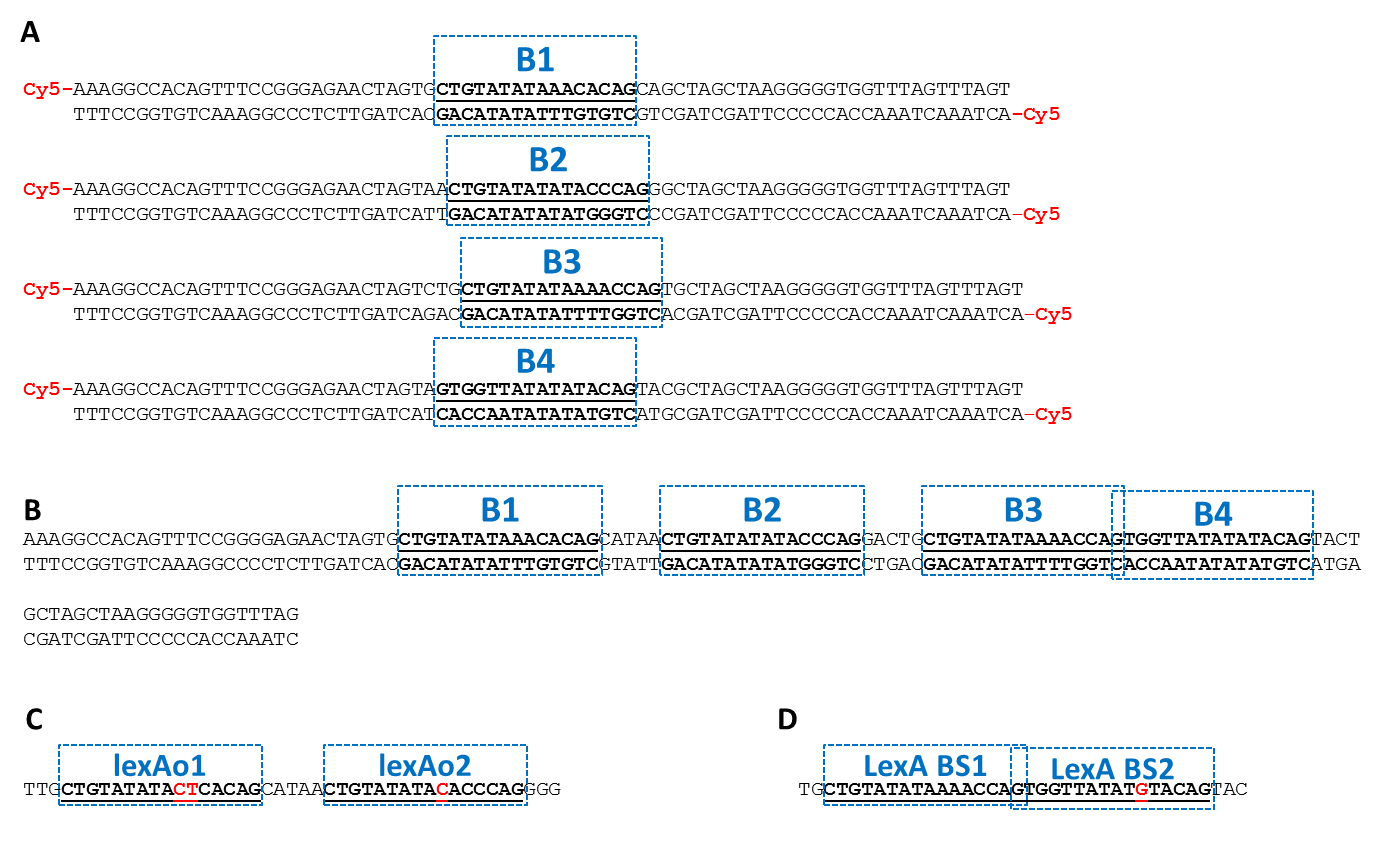


## Figure B: Sequences of the core promoters used in the study


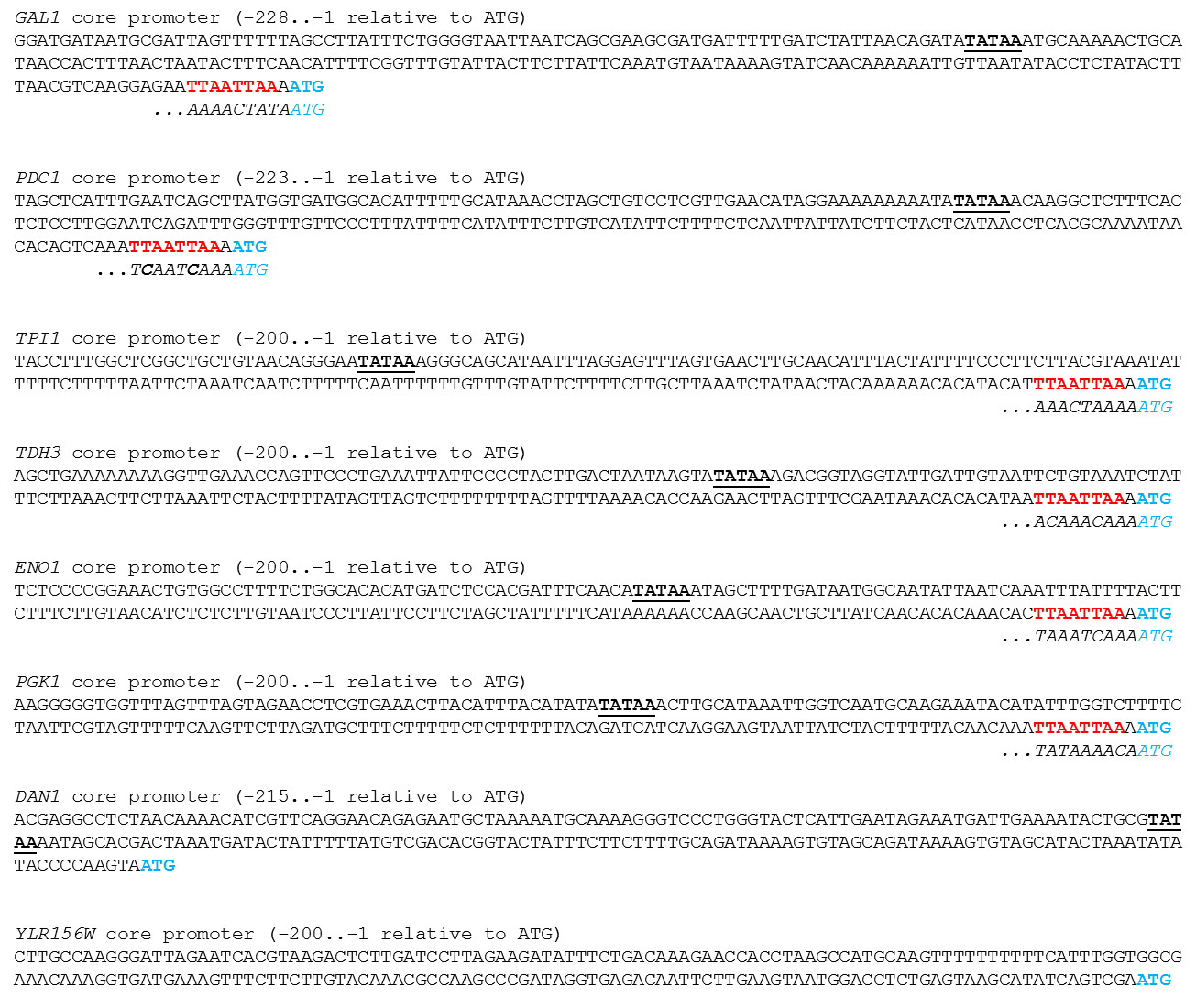
Putative TATA-box is underlined; the PacI restriction site (in red) was introduced at the 3’-ends of the promoters, replacing the original sequence (shown in italics below each modified site). Translation start (ATG) is shown in blue.

## **Figure C:** *In vitro* binding of the LexA binding sites to the purified sTF16 (EMSA)

Similar in-vitro binding efficiency for the purified sTF16 was observed with the four versions of the LexA-binding sites in EMSA. Equal amounts of each Cy-5-labelled DNA probe and the purified sTF were incubated with increasing quantities of an unlabelled DNA competitor containing four LexA-binding sites (B1-B2-B3-B4). In each case, the sTF-labelled-probe complex was partially destabilized in the presence of 2 ng/μl of the unlabelled competitor DNA (+), and almost completely removed in the presence of 20 ng/μl of the competitor (++). The control reactions contained no sTF or competitor.


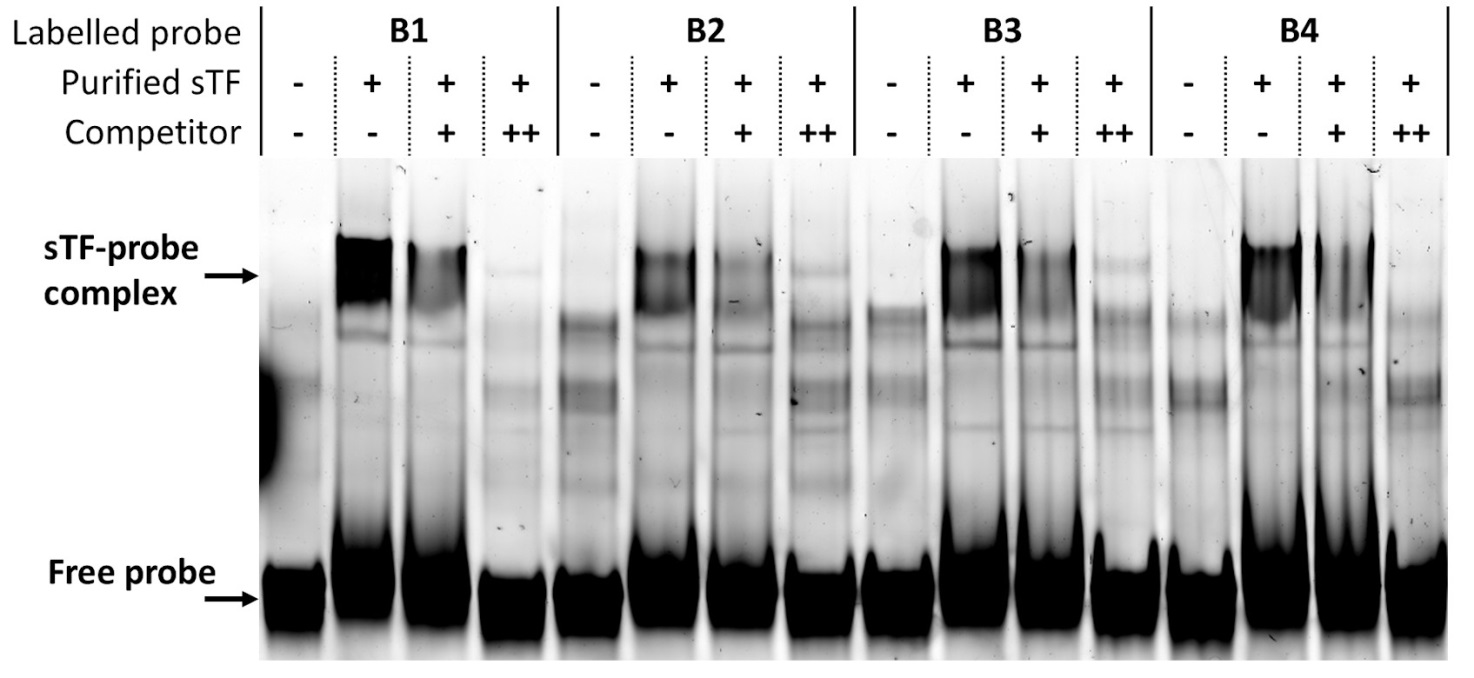


## **Figure D: The** assessment of the amplification gain in the expression systems.

**A)** An input signal, represented by low constitutive transcription activity of the *TDH3*cp controlling the expression of sTFs, is transformed into an output signal of the synthetic promoters controlled by the sTFs. The input signal was estimated from a control experiment, where the mCherry gene was under direct control of the *TDH3*cp (blue line; mCherry signal obtained from the strain carrying pBID1-TTA-6 plasmid as also shown in Fig 2C). The output signal was monitored by the level of mCherry fluorescence for each version of the sTFs and synthetic promoters (also shown in Fig 3). The maximal amplification gain (approximately 38×) was observed in the system composed of the strong sTF16 and the pBID2-EP-8 (green arrow). **B)** An input signal, represented by transcription activity of the *MET17*p controlling the expression of sTFs, can be modulated by the amount of methionine present in the medium. The input signal was estimated from a control experiment, where the mCherry gene was under the direct control of the *MET17*p. The output signal was monitored by the level of mCherry fluorescence for both versions of the sTFs and the synthetic promoter containing 8 BS (also shown in Fig 3). The maximal amplification gain (approximately 10×) was observed in the system with the strong sTF16 in 500μM methionine (green arrow).


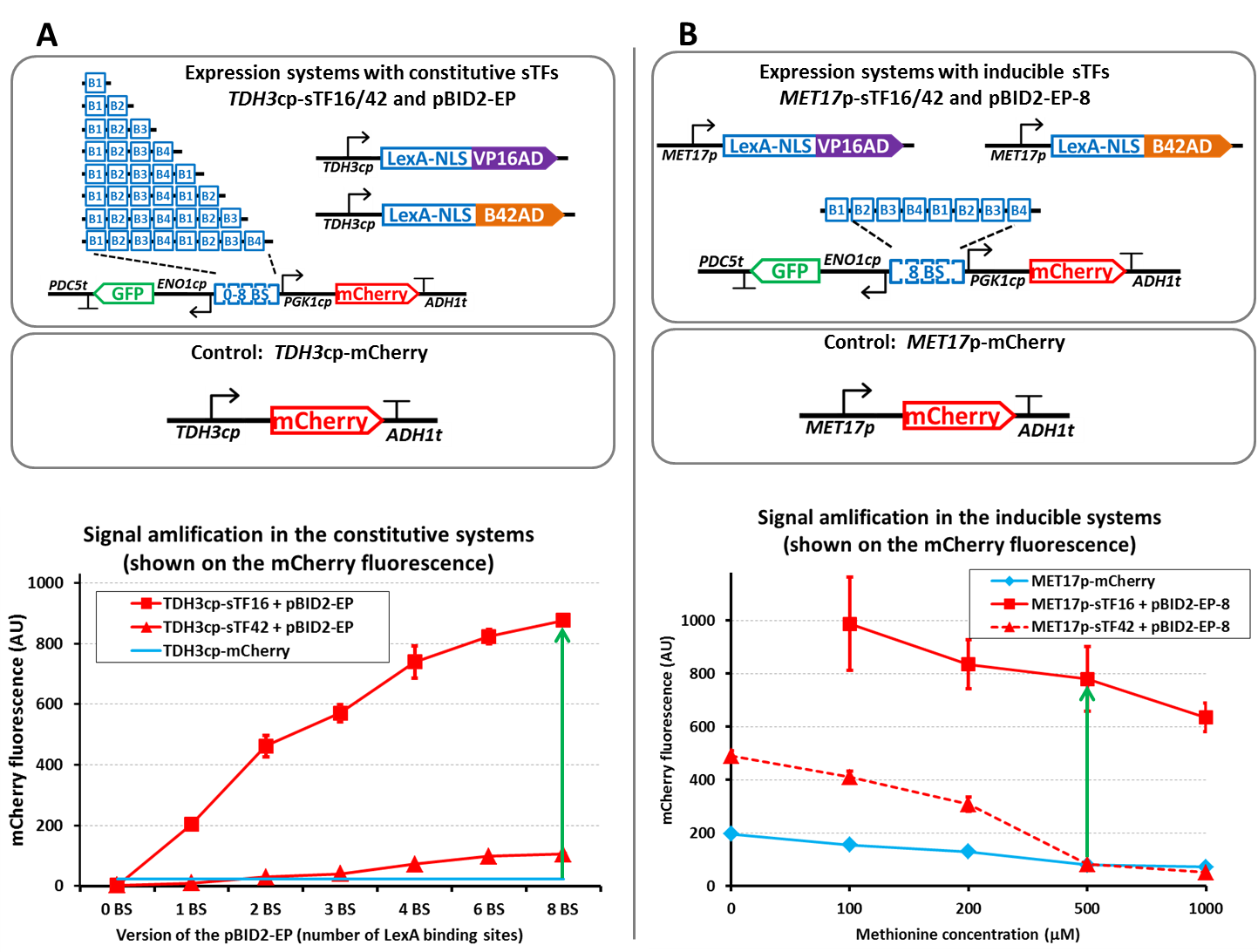


## **Figure E:** Transcription analysis of the constitutive system based on strong sTF16 and pBID2-EP-8, an assessment of the system’s performance in different growth conditions.

The strain was grown in SCD-HU medium for 16 hours (time point 0 hours). The cells were washed and inoculated to OD_(600)_=1.5 in four different media (SCD, SC-EtOH, YPD, and YP-EtOH) for subsequent cultivations. Samples for transcription analysis were collected at the time points specified in the graphs. The expression of the sTF was monitored by the qPCR with primers 1121 and 1122 (specific for LexA), the mCherry expression was monitored by qPCR with primers 890 and 891. The normalization of expression was done by division of the signal for each gene by the corresponding signal of the UBC6 gene (primers 1189 and 1190). UBC6 was used as a control in the transcription analysis, as it confers very consistent expression level in large spectrum of diverse conditions [12]. The values represent the averages from 2 experiments. The amplification of the transcription input signal (as determined by the sTF expression level) into transcription output signal (as determined by the mCherry expression level) calculated from all measured data was 87.6 ± 31.4.


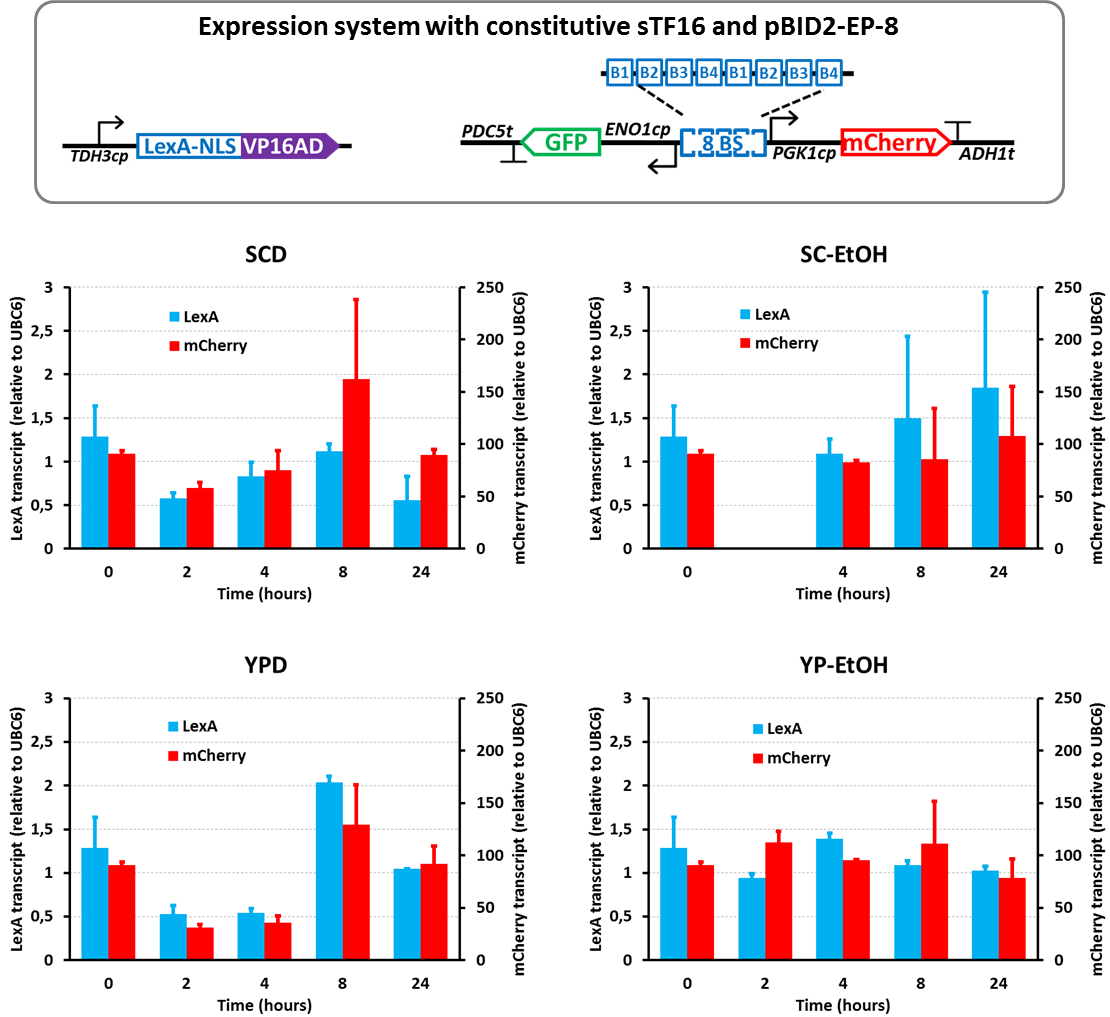


## **Figure F:** The assessment of toxicity of the selected DNA constructs in different concentrations of methionine.

The strains were cultivated in the SCD medium with the indicated concentrations of methionine in a Bioscreen apparatus (Bioscreen C MBR automated turbidometric analyser, Growth Curves Ltd, Finland). The OD measurements were taken every 30 minutes. An identical growth in all tested conditions was observed for the control strain (CEN.PK113-11C), the strains with constitutive sTFs (*TDH3*cp), and the strain with weak inducible sTF-42 (*MET17*p). The strain with strong inducible sTF-16 (*MET17*p) caused a mild growth phenotype in media containing 100-1000μM of methionine, and a significant growth defect in medium without methionine. The graphs represent an average of four independent cultures.


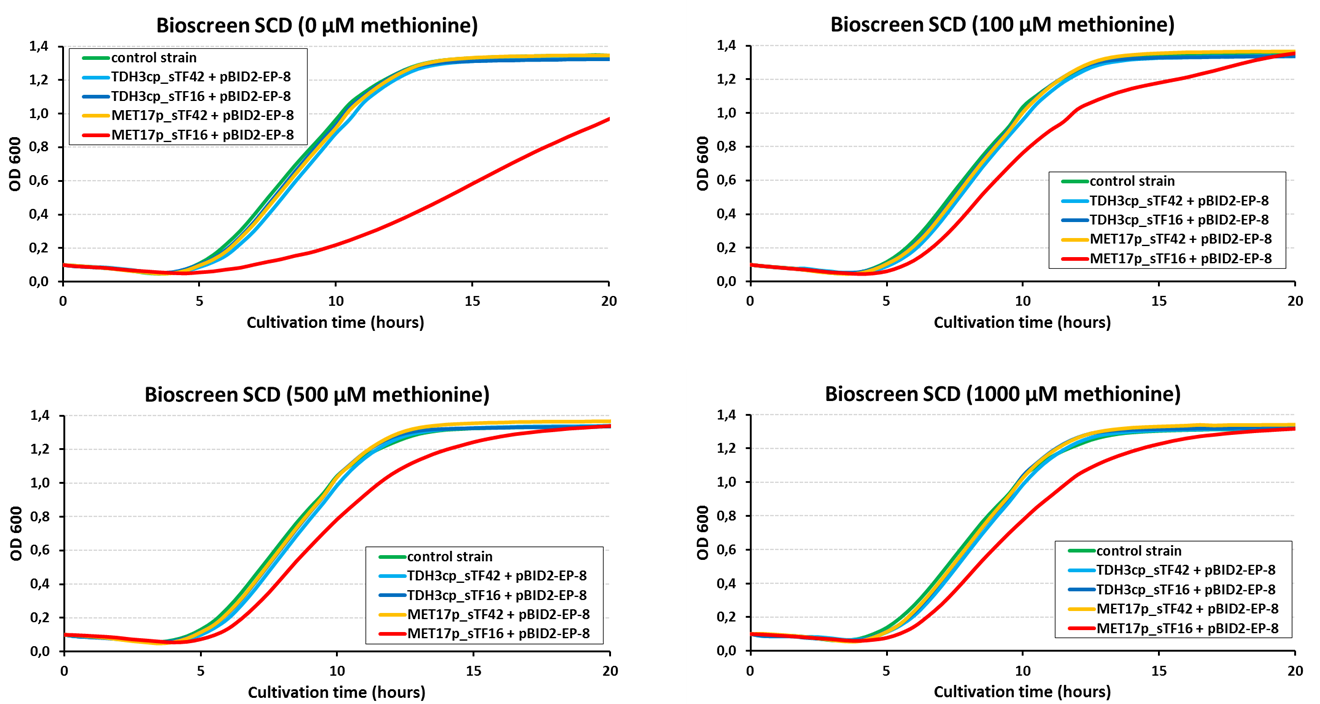


## **Figure G:** *In silico* modeling of the system with strong constitutive sTF16 and pBID2-ED

A hypothesis was tested that there is an additional effect given by the combination of the actual binding sites used in the BS module and the *DAN1* core promoter. We allowed the models corresponding to the constructs with 4 and 8 binding sites to have different kinetic parameters for the association of the core promoter with the polymerase bound to sTF than the other 5 models corresponding to the constructs with 0, 1, 2, 3, and 6 binding sites. The training set for the estimation of the 4 kinetic parameters (association and dissociation kinetics for the core promoter with the polymerase bound to sTF in the 2 models) consisted of the constructs with 1, 4, 6 and 8 boxes. The experimental data from the systems with 0, 2 and 3 boxes were used for model validation.


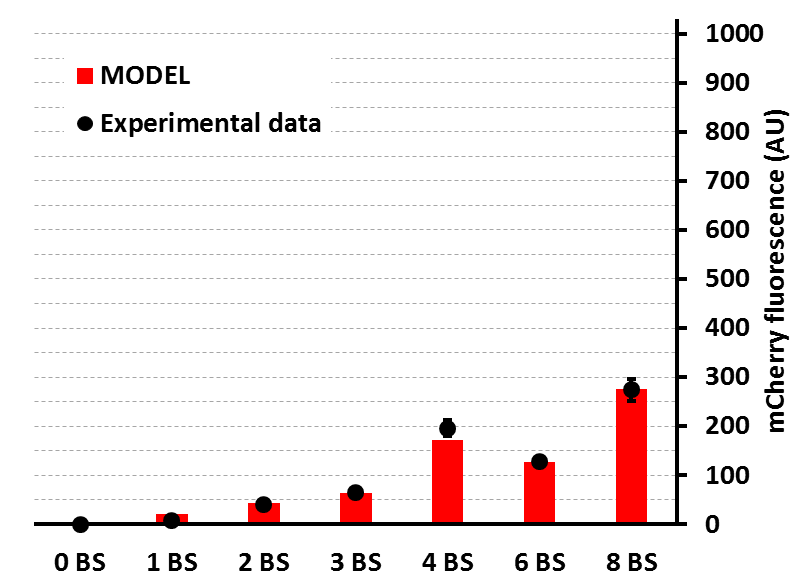


## **Figure H: The residuals distributions for the considered systems**.

**A-D**) The residual distributions for the 4 concentrations of methionine considered for the strong induced system using sTF16. **E)** The residual distribution for the strong constitutive system using sTF16 and pBID2-EP. **F)** The residual distribution for the strong constitutive system using sTF16 and pBID2-ED. **G-K)** The residual distribution for the 5 concentrations of methionine considered for the weak induced system using sTF42. **L)** The residual distribution for the weak constitutive system using sTF42 and pBID2-EP.


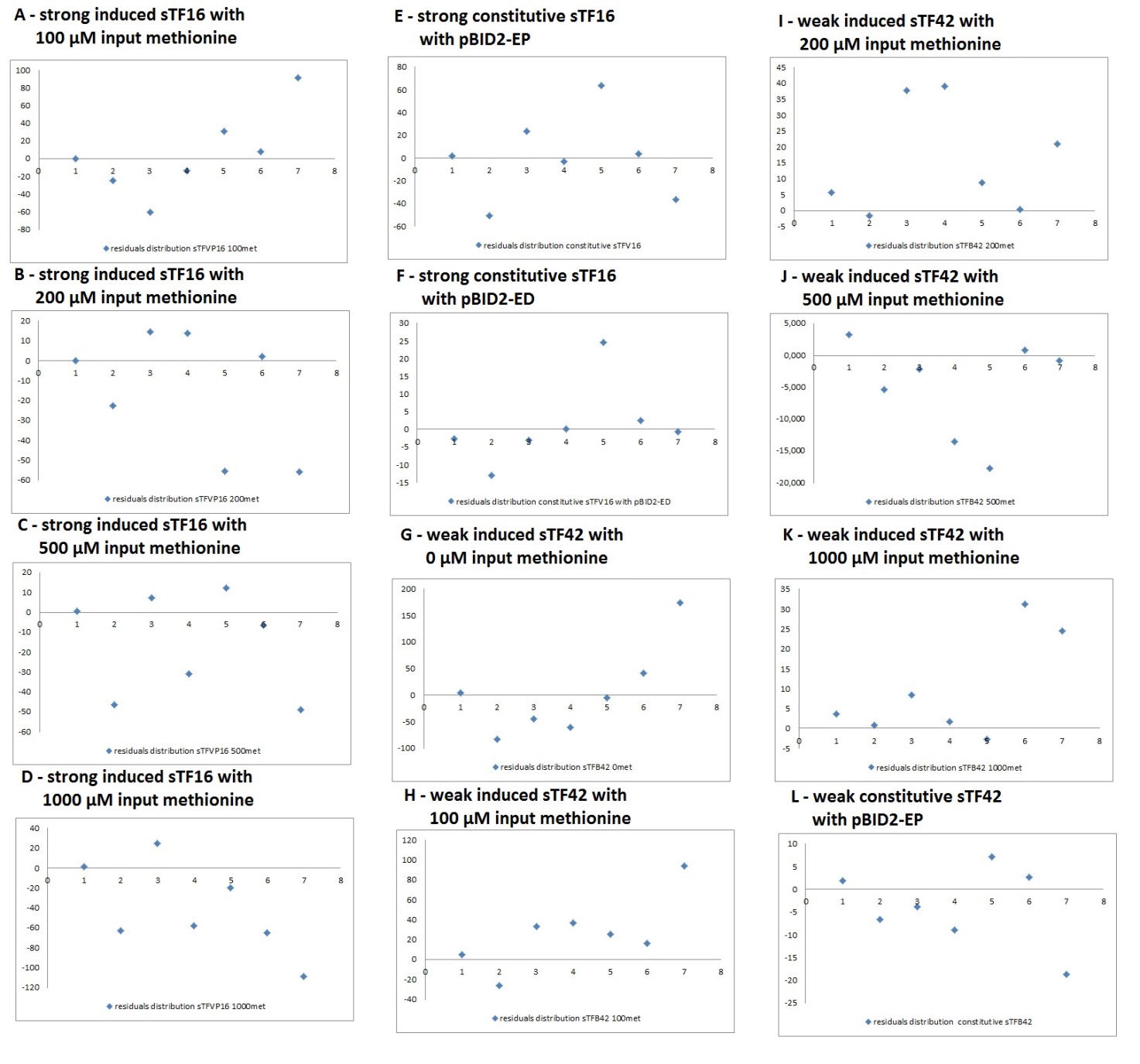


## Table A: List of plasmids

| Plasmid name | marker | Description |
| --- | --- | --- |
| pET26b- sTF16-6H | kanMX | *E. coli* expression plasmid for production of sTF16-6×HIS for EMSA |
| pHIS3i-*Mp*-sTF42 | *HIS3* | Weak version of the sTF (B42AD), expression controlled by the *MET17* p. |
| pHIS3i-*Mp*-sTF16 | *HIS3* | Strong version of the sTF (VP16AD), expression controlled by the *MET17* p. |
| pHIS3i-*Tcp*-sTF42 | *HIS3* | Weak version of the sTF (B42AD), expression controlled by the *TDH3* core p. |
| pHIS3i-*Tcp*-sTF16 | *HIS3* | Strong version of the sTF (VP16AD), expression controlled by the *TDH3* core p. |
| pHIS3i-(wop)-sTF16 | *HIS3* | Strong version of the sTF (VP16AD), control construct without promoter |
| pHIS3i-*Mp*-mCh | *HIS3* | *MET17*p – mCherry; control construct for assessment of input signal |
| pBID1-PGA-6 | *URA3* | *PGK1*cp – mCherry; *GAL1*cp – GFP; 6×LexA binding sites 3×(B1+B2) |
| pBID1-PGB-6 | *URA3* | *PGK1*cp – GFP; *GAL1*cp – mCherry; 6×LexA binding sites 3×(B1+B2) |
| pBID1-TTA-6 | *URA3* | *THD3*cp – mCherry; *TPI1*cp – GFP; 6×LexA binding sites 3×(B1+B2) |
| pBID1-TTB-6 | *URA3* | *THD3*cp – GFP; *TPI1*cp – mCherry; 6×LexA binding sites 3×(B1+B2) |
| pBID1-EPA-6 | *URA3* | *PGK1*cp – mCherry; *ENO1*cp – GFP; 6×LexA binding sites 3×(B1+B2) |
| pBID1-EPB-6 | *URA3* | *PGK1*cp – GFP; *ENO1*cp – mCherry; 6×LexA binding sites 3×(B1+B2) |
| pBID1-PGA-F | *URA3* | *PGK1*p – mCherry; *GAL1*p – GFP |
| pBID1-PGB-F | *URA3* | *PGK1*p – GFP; *GAL1*p – mCherry |
| pBID1-TTA-F | *URA3* | *THD3*p – mCherry; *TPI1*p – GFP |
| pBID1-TTB-F | *URA3* | *THD3*p – GFP; *TPI1*p – mCherry |
| pBID1-EPA-F | *URA3* | *PGK1*p – mCherry; *ENO1*p – GFP |
| pBID1-EPB-F | *URA3* | *PGK1*cp – GFP; *ENO1*cp – mCherry |
| pBID2-EP-0 | *URA3* | *PGK1*cp – mCherry; *ENO1*cp – GFP; 0×LexA binding site |
| pBID2-EP-1 | *URA3* | *PGK1*cp – mCherry; *ENO1*cp – GFP; 1×LexA binding site (B1) |
| pBID2-EP-2 | *URA3* | *PGK1*cp – mCherry; *ENO1*cp – GFP; 2×LexA binding sites (B1+B2) |
| pBID2-EP-3 | *URA3* | *PGK1*cp – mCherry; *ENO1*cp – GFP; 3×LexA binding sites (B1+B2+B3) |
| pBID2-EP-4 | *URA3* | *PGK1*cp – mCherry; *ENO1*cp – GFP; 4×LexA b. sites (B1+B2+B3+B4) |
| pBID2-EP-6 | *URA3* | *PGK1*cp – mCherry; *ENO1*cp – GFP; 6×LexA b. sites (B1+B2+B3+B4+B1+B2) |
| pBID2-EP-8 | *URA3* | *PGK1*cp – mCherry; *ENO1*cp – GFP; 8×LexA b. sites 2×(B1+B2+B3+B4) |
| pBID2-ED-0 | *URA3* | *DAN1*cp – mCherry; *ENO1*cp – GFP; 0×LexA binding site |
| pBID2-ED-1 | *URA3* | *DAN1*cp – mCherry; *ENO1*cp – GFP; 1×LexA binding site (B1) |
| pBID2-ED-2 | *URA3* | *DAN1*cp – mCherry; *ENO1*cp – GFP; 2×LexA binding sites (B1+B2) |
| pBID2-ED-3 | *URA3* | *DAN1*cp – mCherry; *ENO1*cp – GFP; 3×LexA binding sites (B1+B2+B3) |
| pBID2-ED-4 | *URA3* | *DAN1*cp – mCherry; *ENO1*cp – GFP; 4×LexA b. sites (B1+B2+B3+B4) |
| pBID2-ED-6 | *URA3* | *DAN1*cp – mCherry; *ENO1*cp – GFP; 6×LexA b. sites (B1+B2+B3+B4+B1+B2) |
| pBID2-ED-8 | *URA3* | *DAN1*cp – mCherry; *ENO1*cp – GFP; 8×LexA b. sites 2×(B1+B2+B3+B4) |
| pBID2-E156-0 | *URA3* | *YLR156W*cp – mCherry; *ENO1*cp – GFP; 0×LexA binding site |
| pBID2-E156-1 | *URA3* | *YLR156W*cp – mCherry; *ENO1*cp – GFP; 1×LexA binding site (B1) |
| pBID2-E156-2 | *URA3* | *YLR156W*cp – mCherry; *ENO1*cp – GFP; 2×LexA binding sites (B1+B2) |
| pBID2-E156-3 | *URA3* | *YLR156W*cp – mCherry; *ENO1*cp – GFP; 3×LexA binding sites (B1+B2+B3) |
| pBID2-E156-4 | *URA3* | *YLR156W*cp – mCherry; *ENO1*cp – GFP; 4×LexA b. sites (B1+B2+B3+B4) |
| pBID2-E156-6 | *URA3* | *YLR156W*cp – mCherry; *ENO1*cp – GFP; 6×LexA b. sites (B1+B2+B3+B4+B1+B2) |
| pBID2-E156-8 | *URA3* | *YLR156W*cp – mCherry; *ENO1*cp – GFP; 8×LexA b. sites 2×(B1+B2+B3+B4) |

## Table B: List of primers

| number | Sequence 5' -> 3' |
| --- | --- |
| 484 | ACTTTGAACCCAATCATCCA |
| 485 | CACCAACTGCCTTAGTTTCTG |
| 806 | ATATACTAGTGCTGGTATCAATAAAGATATCGAG |
| 807 | ATATACTAGTGGAGGCATCTCCACTCAGCA |
| 858 | CGCGCACATTTCCCCGAAAAGTGCCACCTGGCTGTATATAAACACAGCATAACTGTATATATACCCAGGGATGATAATGCGATTAGTTTTTTAG |
| 861 | CATAAGACAAAGTAGTAACCAAAG |
| 869 | AAGGGAACAAAAGCTGGTACCG |
| 870 | ATATCCCGGGACCTGGCTGTATATAAACACAGC |
| 871 | ATATCCCGGGCACTTTTCGGGGAAATGTGC |
| 872 | CTGGGTATATATACAGTTATGCTG |
| 890 | GTGATGAACTTCGAGGACGG |
| 891 | TTCAGCCTCTGCTTGATCTC |
| 892 | TTTAATTAATTTGACTGTGTTATTTTGCG |
| 893 | TTATGCTGTGTTTATATACAGCCAGGTCCCTAGCTCATTTGAATCAGCTTATG |
| 898 | GAAAAGAAAAAAATTGATCTATCGGAATTGGATCCTCATTACTTGTATAACTCGTCCATGCC |
| 899 | ACGCAAAATAACACAGTCAAATTAATTAAAATGGTGAGCAAGGGCGAGGAG |
| 901 | TTTAATTAATTCTCCTTGACGTTAAAGTATAGAG |
| 902 | GTTCCGCGCACATTTCCCCGAAAAGTGCCCGGGTTTTTTCGAAACGCAGAATTTTCGAG |
| 903 | AATTCCGATAGATCAATTTTTTTCTTTTCTC |
| 904 | ATACTTTAACGTCAAGGAGAATTAATTAAAATGTCTAAAGGTGAAGAATTATTC |
| 905 | TGACATAACTAATTACATGACTCGAGGTCGACGGCTATTATTTGTACAATTCATCCATACCATG |
| 971 | TCGCAACGGCGACTGGCTGGAATTCCCTCCCAAGAAGAAGCGCAAGGTC |
| 972 | CTTATTCAGTTAGCTAGCTGAGCTCNTCAGCCGCCGTACTCGTCAATG |
| 973 | CGAGTTATACAAGTCTAGAACTAGTACGGCCCCCCCCACGGACGTCTC |
| 974 | TCCTGAACAATTGTCCATACTAGTGCCGCCGTACTCGTCAATGCC |
| 1007 | CACAATGTTTACATCATGGCTG |
| 1008 | TCGTTTGGATCTTTGGATAAGG |
| 1009 | TCGCGGATCCTCATTACTTGTATAACTCG |
| 1010 | TATAACTAGTGCTGTATATAAACACAGCAGCTAGCTAAGGGGGTGGTTTAGTTTAGTAG |
| 1011 | TATAACTAGTGCTGTATATAAACACAGCATAACTGTATATATACCCAGGGCTAGCTAAGGGGGTGGTTTAGTTTAGTAG |
| 1012 | TATAACTAGTGCTGTATATAAACACAGCATAACTGTATATATACCCAGGACTGCTGTATATAAAACCAGCTAGCTAAGGGGGTGGTTTAGTTTAGTAG |
| 1013 | ACAGTTTCCGGGGAGAACTAGTGCTGTATATAAACACAGCATAACTGTATATATACCCAGGACTGCTGTATATAAAACCAGTGG |
| 1014 | ACTAAACCACCCCCTTAGCTAGCAGTACTGTATATATAACCACTGGTTTTATATACAGCAGTCC |
| 1017 | ATTACATATGAAAGCGTTAACGGCCAG |
| 1018 | TATAGTCGACGCCGCCGTACTCGTCAATG |
| 1033 | AAGGGAACAAAAGCTGTCGACACTAGTAGCTGAAAAAAAAGGTTG |
| 1034 | TTAACGCTTTCATTGTCTAGATTTAATTAATTATGTGTGTTTATTCG |
| 1043 | AAAGGCCACAGTTTCCGGGAGAACTAGTGCTGTATATAAACACAGCA |
| 1044 | ACTAAACTAAACCACCCCCTTAGCTAGCTGCTGTGTTTATATACAGCA |
| 1045 | AAAGGCCACAGTTTCCGGGAGAACTAGTAACTGTATATATACCCAGG |
| 1046 | ACTAAACTAAACCACCCCCTTAGCTAGCCCTGGGTATATATACAGTTA |
| 1047 | AAAGGCCACAGTTTCCGGGAGAACTAGTCTGCTGTATATAAAACCAGT |
| 1048 | ACTAAACTAAACCACCCCCTTAGCTAGCACTGGTTTTATATACAGCAG |
| 1049 | AAAGGCCACAGTTTCCGGGAGAACTAGTAGTGGTTATATATACAGTAC |
| 1050 | ACTAAACTAAACCACCCCCTTAGCTAGCGTACTGTATATATAACCACT |
| 1051 | AAAGGCCACAGTTTCCGGGGAG |
| 1052 | CTAAACCACCCCCTTAGCTAGC |
| 1078 | CTTCACCTTTAGACATTTTAATTAATTCTCCTTGACGTTAAAGTATAGAGG |
| 1079 | CGCCCTTGCTCACCATTTTAATTAATTTGACTGTGTTATTTTGCGTGAG |
| 1080 | CAGTCGCATGTGTGGAAATGTAAAGAGCCCCA |
| 1081 | CATTTCCACACATGCGACTGGGTGAGCATATG |
| 1082 | CTTCACCTTTAGACATTTTAATTAATTTGACTGTGTTATTTTGCGTGAG |
| 1083 | CGCCCTTGCTCACCATTTTAATTAATTCTCCTTGACGTTAAAGTATAGAGG |
| 1084 | CTTCACCTTTAGACATTTTAATTAAATGTATGTGTTTTTTGTAGTTATAG |
| 1085 | CGCCCTTGCTCACCATTTTAATTAATTATGTGTGTTTATTCGAAACTAAGTTC |
| 1086 | TCTTTGAAATAATAATGCCATTTTTTTGAGTTATAAT |
| 1087 | TGGCATTATTATTTCAAAGAATACGTAAATAATTAATAG |
| 1088 | CTTCACCTTTAGACATTTTAATTAATTATGTGTGTTTATTCGAAACTAAGTTC |
| 1089 | CGCCCTTGCTCACCATTTTAATTAAATGTATGTGTTTTTTGTAGTTATAG |
| 1090 | CTTCACCTTTAGACATTTTAATTAATTTGTTGTAAAAAGTAGATAATTACTTCC |
| 1091 | CGCCCTTGCTCACCATTTTAATTAAGTGTTTGTGTGTTGATAAGCAGTTG |
| 1092 | ATGCTTTCTAGATTCCTGACTTCAACTCAAG |
| 1093 | GTCAGGAATCTAGAAAGCATACTATACTATTCGACAC |
| 1094 | CTTCACCTTTAGACATTTTAATTAAGTGTTTGTGTGTTGATAAGCAGTTG |
| 1095 | CGCCCTTGCTCACCATTTTAATTAATTTGTTGTAAAAAGTAGATAATTACTTCC |
| 1121 | CGATCCTTCCTTATTCAAGCC |
| 1122 | AACAGTTCGACTTTATTGCCC |
| 1123 | ATGGTGAGCAAGGGCGAGG |
| 1124 | ATAAATCATAAGAAATTCGCGGATC |
| 1126 | TAACTGTATATATACCCAGGGCTAGCACGAGGCCTCTAACAAAACATC |
| 1129 | TCCTCGCCCTTGCTCACCATTACTTGGGGTATATATTTAGTATGCTAC |
| 1141 | TAACTGTATATATACCCAGGGCTAGCCTTGCCAAGGGATTAGAATCAC |
| 1144 | TCCTCGCCCTTGCTCACCATTCGACTGATATGCTTACTCAGAGG |
| 1189 | ACTTTCCCGTCTGATTATCCA |
| 1190 | TAATTGATCCTGTCGTGGCT |

## Table C: The biochemical reaction network for the methionine induced sTF system.

|  | **Process** | **Biochemical Reaction** | **Kinetic parameters** |
| --- | --- | --- | --- |
| 1 | **Pol-DTF binding** | Pol + DTF = Pol:DTF | k_PolDTF_binding+; k_PolDTF_binding |
| 2 | **MetTF-BTF binding** | MetTF + BTF = MetTF:BTF | k_MetTF_binding+; k_MetTF_binding |
| 3 | **MeTF-sequestration** | Met + MetTF =Met:MetTF | k_MetTF_sequestration+; k_MetTF_sequestration- |
| 4 | **TF- transcription** | MetTF:BTF + Pol:DTF -> MetTF:BTF + MTFc + Pol + DTF | k_TF_transcription |
| 5 | **TF-translation** | MTFc -> MTFc + TFc | k_TF_translation |
| 6 | **TF translocation** | TFc -> TFn | k_TF_translocation |
| 7 | **TF-DNA binding** | TFn + B = TFn:B | k_TF_binding+; _TF_binding- |
| 8 | **Pol-recruitment** | TFn:B + Pol = Pol:TFn:B | k_Pol_TFn:B_binding+;  k_Pol_ TFn:B_binding- |
| 9 | **CP-binding** | Pol:TFn:B + CP = Pol:TFn:B:CP | k_TFcomplex_CP_binding+;  k_TFcomplex_CP_binding- |
| 10 | **mCherry transcription** | Pol:TFn:B:CP ->TFn:B + Pol + CP + MmCherryc | k_mCherry_transcription |
| 11 | **mCherry translation** | MmCherryc -> mCherryc + MmCherryc | k_ mCherry_translation |
| 12-14 | **Protein degradations** | TFc -> Φ  TFn -> Φ  mCherryc -> Φ | k_TF_degrade  k_TF_degrade  k_protein_degrade |
| 15-16 | **MRNA degradations** | MmCherry_c -> Φ  MTFc -> Φ | k_MRNA_degrade  k_MRNA_degrade |
| 17 | **polymerase binding to CP (in the absence of the boxes )** | Pol + CP = Pol:CP | k_Pol_binding+;  k_Pol_binding- |
| 18 | **Base transcription for mCherry (in the absence of the boxes)** | Pol:CP -> CP + Pol + MmCherryc | k_mCherry_transcription |

## **Table D: The biochemical reaction network for the constitutive sTF system.**

The set of reactions 2-4 from the S3Table corresponding to the methionine-induced transcription of sTF is replaced by reaction 2 corresponding to the constitutive sTF transcription. All the other reactions are identical with the corresponding ones from the S3Table.

|  | **Process** | **Biochemical Reaction** | **Kinetic parameters** |
| --- | --- | --- | --- |
| 1 | **Pol-DTF binding** | Pol + DTF = Pol:DTF | k_PolDTF_binding+; k_PolDTF_binding |
| 2 | **TF- transcription** | Pol:DTF -> MTFc + Pol + DTF | k_TF_transcription |
| 3 | **TF-translation** | MTFc -> MTFc + TFc | k_TF_translation |
| 4 | **TF translocation** | TFc -> TFn | k_TF_translocation |
| 5 | **TF-DNA binding** | TFn + B = TFn:B | k_TF_binding+; k_TF_binding- |
| 6 | **Pol-recruitment** | TFn:B + Pol = Pol:TFn:B | k_Pol_TFn:B_binding+;  k_Pol_ TFn:B_binding- |
| 7 | **CP-binding** | Pol:TFn:B + CP = Pol:TFn:B:CP | k_TFcomplex_CP_binding+;  k_TFcomplex_CP_binding- |
| 8 | **mCherry transcription** | Pol:TFn:B:CP ->TFn:B + CP + Pol + MmCherryc | k_mCherry_transcription |
| 9 | **mCherry translation** | MmCherryc -> mCherryc + MmCherryc | k_ mCherry_translation |
| 10-12 | **Protein degradations** | TFc -> Φ  TFn -> Φ  mCherryc -> Φ | k_TF_degrade  k_TF_degrade  k_protein_degrade |
| 13-14 | **MRNA degradations** | MmCherry_c -> Φ  MTFc -> Φ | k_MRNA_degrade  k_MRNA_degrade |
| 15 | **polymerase binding to CP (in the absence of the boxes)** | Pol + CP = Pol:CP | k_Pol_binding+;  k_Pol_binding- |
| 16 | **Base transcription for mCherry (in the absence of the boxes)** | Pol:CP -> CP + Pol + MmCherryc | k_mCherry_transcription |

## Table E: Cellular localization of the components included in the mathematical model

| **Reactant** | **Cellular localization** | **Reactant** | **Cellular localization** |
| --- | --- | --- | --- |
| Pol | nucleus | TFc | cytoplasm |
| DTF | nucleus | TFn | nucleus |
| Pol:DTF | nucleus | B | nucleus |
| MTFc | cytoplasm | TFn:B | nucleus |
| MetTF | nucleus | CP | nucleus |
| Met | nucleus | Pol:TFn:B | nucleus |
| BTF | nucleus | Pol:TFn:B:CP | nucleus |
| MetTF:BTF | nucleus | MmCherryc | cytoplasm |
| Met:MetTF | nucleus | mCherryc | cytoplasm |
| Pol:CP | nucleus |  |  |

## Table F: Model parameters values

| **Parameters** | **Value** | **Unit** | **Additional information** |
| --- | --- | --- | --- |
| k_PolDTF_binding+; | 4988.37 | $\frac{pl}{(\#*s)}$ | the same value was used in all models |
| k_PolDTF_binding | 0.192182 | $\frac{1}{s}$ | the same value was used in all models |
| k_MetTF_binding+; | 0.444491 | $\frac{pl}{(\#*s)}$ | the same value was used in all models |
| k_MetTF_binding- | 125.751 | $\frac{1}{s}$ | the same value was used in all models |
| k_MetTF_sequestration+; | 0.738117 | $\frac{pl}{(\#*s)}$ | the same value was used in all models |
| k_MetTF_sequestration- | 0.622979 | $\frac{1}{s}$ | the same value was used in all models |
| k_TF_transcription | 1378.48 | $\frac{{pl}^{2}}{(\#*s)}$ | for the methionine induced sTF systems |
| k_TF_transcription | 2.04445 | $\frac{pl}{s}$ | for the constitutive sTF systems |
| k_TF_translation | 97.5615 | $\frac{1}{s}$ | the same value was used in all models |
| k_TF_translocation | 0.86696 | $\frac{pl}{s}$ | the same value was used in all models |
|  |  |  |  |
| k_TF_binding+; | 0.189786 | $\frac{pl}{(\#*s)}$ | the same value was used in all models |
| k_TF_binding- | 236.853 | $\frac{1}{s}$ | the same value was used in all models |
| k_Pol_TFn:B_binding+; | 6.66667 | $\frac{pl}{(\#*s)}$ | for the systems using sTF^VP16^ |
| k_Pol_ TFn:B_binding- | 74458.6 | $\frac{1}{s}$ | for the systems using sTF^VP16^ |
| k_Pol_TFn:B_binding+; | 0.223382 | $\frac{pl}{(\#*s)}$ | for the systems using sTF^B42^ |
| k_Pol_ TFn:B_binding- | 53400 | $\frac{1}{s}$ | for the systems using sTF^B42^ |
| k_TFcomplex_CP_binding+; | 13.2785 | $\frac{pl}{(\#*s)}$ | for all systems using the pBID-EP |
| k_TFcomplex_CP_binding- | 1.14573e-05 | $\frac{1}{s}$ | for all systems using the pBID-EP |
| k_TFcomplex_CP_binding+; | 0.6129 | $\frac{pl}{(\#*s)}$ | for all systems using the pBID2-ED |
| k_TFcomplex_CP_binding- | 342.944 | $\frac{1}{s}$ | for all systems using the pBID2-ED |
| k_TFcomplex_CP_binding+; | 0.419106 | $\frac{pl}{(\#*s)}$ | for the systems with 0,1,2,3, and 6 sTF-specific binding boxes using the alternative pBID2-ED |
| k_TFcomplex_CP_binding- | 106.621 | $\frac{1}{s}$ | for the systems with 0,1,2,3, and 6 sTF-specific binding boxes using the alternative pBID2-ED |
| k_TFcomplex_CP_binding+; | 0.733533 | $\frac{pl}{(\#*s)}$ | for the systems with 4 and 8 sTF-specific binding boxes using the alternative pBID2-ED |
| k_TFcomplex_CP_binding- | 1.67783e-06 | $\frac{1}{s}$ | for the systems with 4 and 8 sTF-specific binding boxes using the alternative pBID2-ED |
| k_mCherry_transcription | 62.3403 | $\frac{pl}{s}$ | the same value was used in all models |
| k_ mCherry_translation | 31.1701 | $\frac{1}{s}$ | the same value was used in all models |
| k_TF_degrade | 358.685 | $\frac{1}{s}$ | for the systems using sTF^B42^ |
| k_TF_degrade | 226.008 | $\frac{1}{s}$ | for the systems using sTF^VP16^ |
| k_protein_degrade | 12.2269 | $\frac{1}{s}$ | the same value was used in all models |
| k_MRNA_degrade | 863.907 | $\frac{1}{s}$ | the same value was used in all models |
| k_Pol:CP_binding+; | 8.7019e-06 | $\frac{pl}{s}$ | the same value was used in all models |
| k_Pol:CP_binding- | 744.519 | $\frac{1}{s}$ | the same value was used in all models |
| mCherry fluorescence scaling factor | 9.74452 |  | for the methionine induced systems using sTF^VP16^ |
| mCherry fluorescence scaling factor | 4.96371 |  | for the methionine induced systems using sTF^B42^ |
| mCherry fluorescence scaling factor | 10.889 |  | for the constitutive systems using sTF^VP16^ |
| mCherry fluorescence scaling factor | 8.93741 |  | for the constitutive systems using sTF^B42^ |
| mCherry fluorescence scaling factor | 19.48 |  | for the constitutive system using sTF^VP16^ and the alternative pBID2-ED |
| methionine level at 16h time point when starting with 0µM | 3145695 | $\frac{\#}{pl}$ | for all methionine-induced systems receiving 0µM initial methionine concentration and binding boxes varying between 0 and 8 |
| methionine level at 16h time point when starting with 100µM | 12924188 | $\frac{\#}{pl}$ | for all methionine-induced systems receiving 100µM initial methionine concentration and binding boxes varying between 0 and 8 |
| methionine level at 16h time point when starting with 200µM | 27010394 | $\frac{\#}{pl}$ | for all methionine-induced systems receiving 200µM initial methionine concentration and binding boxes varying between 0 and 8 |
| methionine level at 16h time point when starting with 500µM | 208320450 | $\frac{\#}{pl}$ | for all methionine-induced systems receiving 500µM initial methionine concentration and binding boxes varying between 0 and 8 |
| methionine level at 16h time point when starting with 1000µM | 499999999 | $\frac{\#}{pl}$ | for all methionine-induced systems receiving 1000µM initial methionine concentration and binding boxes varying between 0 and 8 |

## Table G: $\boldsymbol{R}^{\boldsymbol{2}}$values measuring the goodness of fit between model predictions and experimental measurements.

| **Model** | $\boldsymbol{R}^{\boldsymbol{2}}$**measure** |
| --- | --- |
| Induced system using sTF16 with 100µM input methionine | 0,980 |
| Induced system using sTF16 with 200 µM input methionine | 0,987 |
| Induced system using sTF16 with 500 µM input methionine | 0,988 |
| Induced system using sTF16 with 1000 µM input methionine | 0,927 |
| Constitutive system using sTF16 | 0,987 |
| Constitutive system using sTF16 and the alternative pBID2-ED illustrated in Figure G | 0,987 |
| Constitutive system using sTF42 | 0,946 |
| Induced system using sTF42 with 0 µM input methionine | 0,834 |
| Induced system using sTF42 100 µM input methionine | 0,904 |
| Induced system using sTF42 200 µM input methionine | 0,941 |
| Induced system using sTF42 500 µM input methionine | 0,872 |
| Induced system using sTF42 1000 µM input methionine | 0,478 |

## References

1. Valkonen M, Mojzita D, Penttila M, Bencina M (2013) Non-invasive High-Throughput Single-Cell Analysis of the Intracellular pH of Yeast by Ratiometric Flow Cytometry. Appl Environ Microbiol.

2. Niedenthal RK, Riles L, Johnston M, Hegemann JH (1996) Green fluorescent protein as a marker for gene expression and subcellular localization in budding yeast. Yeast 12: 773-786.

3. Sikorski RS, Hieter P (1989) A system of shuttle vectors and yeast host strains designed for efficient manipulation of DNA in *Saccharomyces cerevisiae*. Genetics 122: 19-27.

4. Chelliah V, Juty N, Ajmera I, Ali R, Dumousseau M, et al. (2015) BioModels: ten-year anniversary. Nucleic Acids Res 43: D542-548.

5. DiStefano III J (2013) Dynamic Systems Biology Modeling and Simulation: ACADEMIC PRESS.

6. Kirkpatrick S, Gelatt CD, Jr., Vecchi MP (1983) Optimization by simulated annealing. Science 220: 671-680.

7. Hoops S, Sahle S, Gauges R, Lee C, Pahle J, et al. (2006) COPASI- A COmplex PAthway SImulator. Bioinformatics 22: 3067-3074.

8. Costa KD, Kleinstein SH, Hershberg U (2011) Biomedical Model Fitting and Error Analysis. Science Signaling 4.

9. Lobemeier ML (2000) Linearization plots: Time for progress in regression. HMS Beagle.

10. Wertman KF, Mount DW (1985) Nucleotide sequence binding specificity of the LexA repressor of Escherichia coli K-12. J Bacteriol 163: 376-384.

11. Estojak J, Brent R, Golemis EA (1995) Correlation of two-hybrid affinity data with *in vitro* measurements. Mol Cell Biol 15: 5820-5829.

12. Teste MA, Duquenne M, Francois JM, Parrou JL (2009) Validation of reference genes for quantitative expression analysis by real-time RT-PCR in *Saccharomyces cerevisiae*. BMC Mol Biol 10: 99.
